# EPheClass: ensemble-based phenotype classifier from 16S rRNA gene sequences

**DOI:** 10.3389/fbinf.2025.1514880

**Published:** 2025-09-30

**Authors:** Lara Vázquez-González, Carlos Peña-Reyes, Alba Regueira-Iglesias, Carlos Balsa-Castro, Inmaculada Tomás, María J. Carreira

**Affiliations:** 1 Centro Singular de Investigación en Tecnoloxías Intelixentes (CiTIUS), Universidade de Santiago de Compostela, Santiago de Compostela, Spain; 2 Instituto de Investigación Sanitaria de Santiago de Compostela (IDIS), Santiago de Compostela, Spain; 3 School of Management and Engineering Vaud (HES-SO), University of Applied Sciences and Arts Western Switzerland Vaud, Yverdon-lesBains, Switzerland; 4 CI4CB—Computational Intelligence for Computational Biology, SIB—Swiss Institute of Bioinformatics, Lausanne, Switzerland; 5 Oral Sciences Research Group, Special Needs Unit, Department of Surgery and Medical Surgical Specialities, School of Medicine and Dentistry, Universidade de Santiago de Compostela, Santiago de Compostela, Spain; 6 Departamento de Electrónica e Computación, Escola Técnica Superior de Enxeñaría, Universidade de Santiago de Compostela, Santiago de Compostela, Spain

**Keywords:** microbiome, phenotype classification, 16S rRNA gene, machine learning, feature selection, ensemble-based classification

## Abstract

One area of bioinformatics that is currently attracting particular interest is the classification of polymicrobial diseases using machine learning (ML), with data obtained from high-throughput amplicon sequencing of the 16S rRNA gene in human microbiome samples. The microbial dysbiosis underlying these types of diseases is particularly challenging to classify, as the data is highly dimensional, with potentially hundreds or even thousands of predictive features. In addition, the imbalance in the composition of the microbial community is highly heterogeneous across samples. In this paper, we propose a curated pipeline for binary phenotype classification based on a count table of 16S rRNA gene amplicons, which can be applied to any microbiome. To evaluate our proposal, raw 16S rRNA gene sequences from samples of healthy and periodontally affected oral microbiomes that met certain quality criteria were downloaded from public repositories. In the end, a total of 2,581 samples were analysed. In our approach, we first reduced the dimensionality of the data using feature selection methods. After tuning and evaluating different machine learning (ML) models and ensembles created using Dynamic Ensemble Selection (DES) techniques, we found that all DES models performed similarly and were more robust than individual models. Although the margin over other methods was minimal, DES-P achieved the highest AUC and was therefore selected as the representative technique in our analysis. When diagnosing periodontal disease with saliva samples, it achieved with only 13 features an F1 score of 0.913, a precision of 0.881, a recall (sensitivity) of 0.947, an accuracy of 0.929, and an AUC of 0.973. In addition, we used EPheClass to diagnose inflammatory bowel disease (IBD) and obtained better results than other works in the literature using the same dataset. We also evaluated its effectiveness in detecting antibiotic exposure, where it again demonstrated competitive results. This highlights the importance and generalisation aspect of our classification approach, which is applicable to different phenotypes, study niches, and sample types. The code is available at https://gitlab.citius.usc.es/lara.vazquez/epheclass.

## 1 Introduction

One area of bioinformatics that has attracted particular interest in recent years is the classification of diseases using machine learning (Asgari et al., 2018; Zhao et al., 2021). In particular, pathologies caused by an imbalance in the composition of the microbial community (dysbiosis), which are more difficult to predict because there is no specific bacterium to blame (Relvas et al., 2021).

The most commonly used genetic marker in this type of analysis is the 16S ribosomal RNA (rRNA) gene (Rajendhran and Gunasekaran, 2011), which is present in all bacteria and contains both conserved and hypervariable regions. The former are regions that are identical or similar in nucleic acids across bacterial species, and the latter have considerable sequence diversity between different bacterial species. The conserved regions are often used to bind the primer pairs that allow amplification and subsequent sequencing of part or all of the 16S rRNA gene. Meanwhile, the hypervariable regions between the previously fixed primer pairs provide the information needed to find, separate, and count the different bacterial species present in the microbiome being analysed.

In the literature, 16S rRNA gene sequences are often clustered into Operational Taxonomic Units (OTUs), which group sequences based on a defined threshold of sequence similarity and serve as standard units in marker gene analysis. However, OTUs have some limitations, including limited reusability and a lack of comprehensiveness, which can negatively impact the quality of results. An alternative approach is to use k-mers (substrings of nucleotide sequences of length k); however, these suffer from poor interpretability, as individual k-mers do not carry any inherent biological meaning. These limitations can be overcome by using Amplicon Sequence Variants (ASVs), which are any of the derived single DNA sequences obtained from a high-throughput analysis (Callahan et al., 2017).

Machine learning algorithms, such as random forest (RF) or support vector machines (SVM), and neural network algorithms, such as multilayer perceptron (MLP), have been used in various studies to classify patients as healthy or diseased based on their microbiome composition (Uddin et al., 2019; Yu et al., 2022). Several works in the literature classify periodontal disease (Lundmark et al., 2019; Narita and Kodama, 2022; Na et al., 2020; Chen et al., 2021). However, none of these studies employ a rigorous procedure for making reliable and accurate predictions. Such a procedure would require a sufficiently large sample size, data set splitting, and cross-validation, as well as an adequate number of features to support generalisable conclusions.

In this paper, we consider the importance of following a curated pipeline for phenotype classification using 16S rRNA gene amplicon count tables. Furthermore, we aim to achieve the best possible results, ensuring reproducibility and reducing the computational costs. We selected a wide range of popular machine learning algorithms, from classic to newer and more complex: k-nearest neighbours (kNN), RF, SVM, extreme gradient boosting (XGBoost), and MLP, and used them to build dynamic ensemble models.

## 2 Methods

### 2.1 Periodontal disease dataset

This study used a periodontal disease (PD) dataset compiled by our research team. It was assembled from other datasets found in the literature, considering only those that were available in public repositories and that also had metadata associated to properly differentiate the samples and their corresponding pathologies. The study examines the salivary and plaque microbiota of adult patients with varying periodontal health conditions. The V3-V4 region of the 16S rRNA gene was targeted, and Illumina sequencing technology was used. For a summary of the Bioprojects used, refer to Supplementary Table S1.

This dataset comprises multiple independent studies, each with samples containing paired-end sequences ranging from 250 to 300 base pairs (bp). We merged these sequences into contigs with a minimum overlap of 20 bp. Samples that did not meet the minimum number of 5,000 sequences per sample were excluded.

A total of 2,581 samples were collected from various sources. The samples can be classified based on several variables, with the most significant being the type of disease (periodontitis or gingivitis) and the site of sample collection (subgingival plaque, supragingival plaque, or saliva).

Different combinations of variable types were considered, resulting in various subsets of the dataset. To introduce heterogeneity, samples of periodontitis were combined with samples of gingivitis, which is considered an early stage of periodontal involvement. Given the importance of dental plaque in the pathogenesis of periodontitis, the subgingival and supragingival samples were analysed together, while the saliva samples were studied separately. Ultimately, we generated four subsets. Two of these subsets were fairly balanced, while the remaining two were highly unbalanced, as shown in Table 1.

**TABLE 1:** Subsets generated from the original periodontal disease dataset to evaluate EPheClass. Legend: Disease (P: periodontitis, G: gingivitis), #D: number of samples affected by the disease, #Not-D: number of samples not affected by the disease.

Subset	Disease	Collection site	#Samples	#D	#Not-D
PD_s	P	saliva	797	314	483
PGD_s	P + G	saliva	815	332	483
PD_p	P	plaque^a^	1,667	1,298	369
PGD_p	P + G	plaque^a^	1,766	1,397	369

^a^ Subgingival + supragingival samples.

The samples undergo a quality control process using USEARCH (Edgar, 2010). Sequences with a maximum number of expected errors greater than one were excluded (Edgar and Flyvbjerg, 2015), as this threshold, which is based on the sum of base-wise error probabilities, reflects overall sequence quality. In addition, sequences that did not exceed the minimum length of 300 bp were excluded.

Following quality control, we performed additional processing to obtain the ASVs and their abundance table using mothur (Schloss et al., 2009), which is detailed in the Supplementary Section S1. A total of 10,577 ASVs were identified. These tables can be found in https://github.com/Oral-Sciences-Research-Group/Epheclass_dataset.

### 2.2 EPheClass phenotype classification pipeline

**FIGURE 1:**
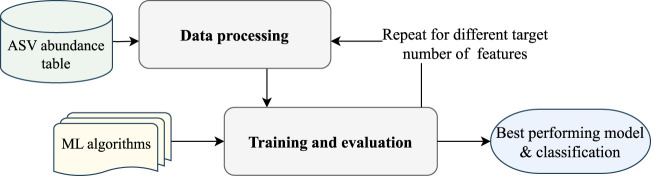
EPheClass pipeline for ensemble-based phenotype classification from ASV abundance tables.

The EPheClass pipeline is proposed for classifying phenotypes, such as diseases. This pipeline, developed in Python 3.10, is shown in Figure 1 and begins with an ASV abundance table containing the frequencies of the ASVs in the different samples, which are then classified into the desired categories. In this proposal, the samples are classified as either affected by the disease (D), or not affected by the disease (Not-D). The pipeline consists of two main modules: data processing and training and evaluation. The data processing step prepares the ASV abundance table for the classification step by selecting the most relevant features to reduce dimensionality and discard ASVs without significance. The training and evaluation step focuses on training the tuned models and subsequently evaluating them to propose the best ensemble model as output.

#### 2.2.1 EPheClass module 1: data processing

**FIGURE 2:**
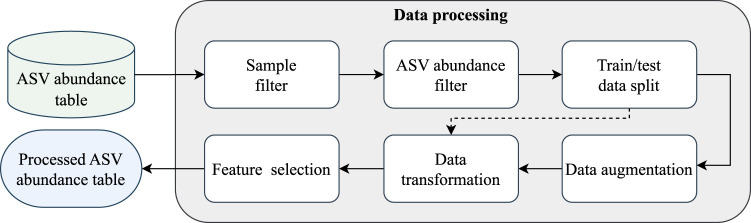
EPheClass pipeline module 1: data processing.

In this first module of the pipeline, shown in Figure 2, the sequences are prepared for the classification module through six steps: sample filtering, ASV abundance filtering, train/test data splitting, data augmentation (optional), data transformation, and feature selection.

In the first step, samples with low counts of ASVs are excluded, and only those with a significant number of sequences are retained. We chose a threshold of at least 2,500 total counts per sample. From the initial 2,581 samples, 42 were discarded.

In the second step, a pseudo count of 1 is added to each value in the abundance table to correct its sparsity. Then, the relative frequencies of the ASVs are calculated for each sample, and the 1,500 most abundant are selected. Thus, we avoid the instability associated with non-abundant ASVs by discarding those with low frequencies (Jiang et al., 2021). Moreover, from a clinical perspective, higher abundance ASVs are often considered more relevant indicators of microbial community shifts and disease states (Nikodemova et al., 2023).

The dataset is then split into training and test sets in a stratified fashion to maintain the same proportions of examples in each class as observed in the original dataset. For the training set, 70% of the data was used. For the test set, the remaining 30% of the data was used. These proportions were chosen considering that 10% of the training subset should be used as the validation set during the 10-fold cross-validation in the model’s tuning process.

Next, an optional data augmentation step is available if the data is significantly unbalanced. Compositional CutMix (Gordon-Rodriguez et al., 2022) was chosen as the data augmentation algorithm because it was specifically designed for compositional data (CoDa), such as ASV count tables. This algorithm creates additional data points by combining pairs of training samples from the same class using complementary subcompositions and renormalisation. The use of cross-validation means that only training data needs to be augmented separately in each iteration, using a fixed random seed to ensure consistency in the augmented data, which is then combined to form the new augmented subset.

Due to the compositional nature of the information (Gloor et al., 2017), we can only work in the CoDa space (also known as simplex) or transform the data into the Euclidean space using a log ratio transformation (Aitchison, 1982). In this case, the centred log-ratio transformation (CLR) was chosen to transform each row (sample) individually, as recommended when working with compositional data (Quinn et al., 2019).

The training data underwent a feature selection (FS) stage to reduce the number of features (ASVs in this case), lower computational costs, and enhance the performance of predictive models. Recursive Feature Elimination (RFE) was employed as the selection method, using three different estimators to prevent any bias in the classification algorithm: RF, SVM, and Logistic Regression (LR). This technique iteratively removes features from the feature set and evaluates the performance of the selected model (estimator) on the reduced feature set. The features with the least impact on the model’s performance are then discarded.

To ensure the extraction of the most selected and likely best features, we chose ASVs that were selected by all three feature selection methods. As a result, we obtained a reduced ASV abundance table at the end of the EPheClass module 1, containing only the most representative ASVs based on the target number of features. As there were *n* different target numbers of features evaluated, *n* different ASV abundance tables were obtained. These tables were then used to determine the best classification model and target number of features.

#### 2.2.2 EPheClass module 2: training and evaluation

**FIGURE 3:**
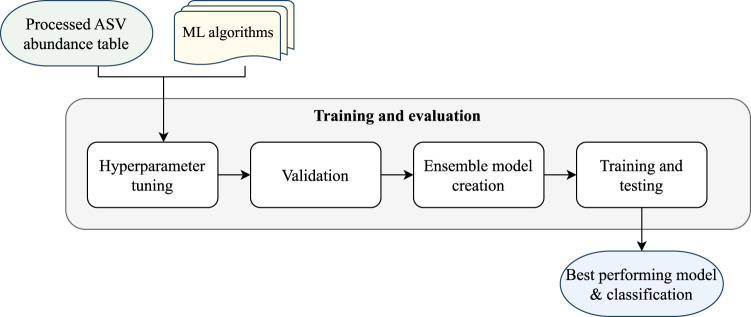
EPheClass pipeline module 2: training and evaluation.

Module 2 of the pipeline, as illustrated in Figure 3, performs the classification and evaluation through four steps: hyperparameter tuning, validation of individual models, creation of ensemble models, and final training and testing of the models.

Firstly, to tackle our classification problem, we need to carry out a process of training and evaluating different models. This should range from traditional techniques such as kNN, RF, or SVM to more complex ones such as XGBoost or MLP. The selection of techniques is heterogeneous due to their fundamental differences, except for XGBoost and RF, which are both decision tree-based. The objective is to develop an ensemble model that surpasses the individual models and overcomes their limitations.

The initial stage involves selecting parameters for each technique, known as hyperparameter tuning. To achieve this, we utilised the *GridSearchCV* function from the Scikit-learn library (Pedregosa et al., 2011). We exhaustively tested various values for several parameters of each algorithm, as shown in the Supplementary Table S2, and then selected the best-performing combinations using 10-fold stratified cross-validation. We chose the most frequently tuned parameters, as well as additional parameters that could have a significant impact on the results.

Although some subsets are more balanced than others, they generally tend to have some degree of imbalance. Therefore, we used the ROC AUC value, hereafter referred to simply as AUC, as the key metric to select the best combination of hyperparameters. In this case, both false negatives and false positives are damaging, as the patient would be misdiagnosed and the disease ignored, or the patient could be subjected to unnecessary treatment. As the AUC distinguishes between positive and negative classes across multiple classification thresholds, it was considered more appropriate for this study.

Figure 3 shows that, after tuning the hyperparameters for each model, we evaluated them individually using stratified 10-fold cross-validation. Next, we employed four different Dynamic Ensemble Selection (DES) techniques (Cruz et al., 2018b) to create promising ensemble models. These techniques dynamically select the best ensemble from a pool of base models for each input. Four techniques from the *DESlib* Python library (Cruz et al., 2018a) were tested: Dynamic Ensemble Selection-Performance (DES-P), Dynamic Ensemble Selection-Clustering (DES-C), k-Nearest Oracles Eliminate (KNORA-E), and k-Nearest Oracles Union (KNORA-U). The DES-P method selects base models that perform better than a random classifier in a domain of competence estimated using kNN (finding the k closest samples to the input). The DES-C method selects base models based on their accuracy and diversity, using the double error diversity measure. The KNORA-E method selects base models that perform perfectly on samples in the region of competence, reducing this region if necessary to find perfect models. The KNORA-U method selects base models that correctly classify at least one sample in the region of competence, with each model being assigned a weight according to the number of correct classifications.

Hyperparameter tuning is performed to select the optimal value for parameter *k*, which determines the region of competence in dynamic ensembles. This is done using the already tuned and pre-trained individual models. Evaluation is then carried out using cross-validation, and individual models and ensemble models are finally evaluated again through training and testing processes.

Finally, overall performance was visualised using ROC AUC plots. Statistical comparisons were then conducted using the Venkatraman test: the paired version (Venkatraman, 1996) was used to compare models within the same data partition, while the unpaired version (Venkatraman, 2000) was applied to assess differences across data partitions or between class imbalance strategies.

### 2.3 Application of EPheClass

#### 2.3.1 Diagnosing periodontal disease (PD)

The PD dataset was analysed using this pipeline to ensure the quality, robustness, and reproducibility of the results. A series of experiments were conducted to identify the model that would achieve the best results while prioritising a smaller number of features.

Four subsets of the PD dataset (see Table 1) were assessed: PD_p, PGD_p, PD_s, and PGD_s. We evaluated different numbers of features for each subset, ranging from 2 to 15, focusing on lower values to thoroughly test the feasibility of dimensionality reduction. Experiments were conducted for each combination of subset and number of features.

The EPheClass pipeline was used for each experiment. To address the strong imbalance in the classes, data augmentation was performed on the healthy samples of the training set for the two plaque subsets. This was done to achieve the same number of healthy and diseased samples. As the plaque subsets consisted of samples from both supragingival and subgingival sites, we separated the samples in each fold based on their collection site and augmented them separately. Following this step, the PD_p subset was augmented by 630 healthy samples, and the PGD_p subset was augmented by 690 healthy samples. In the second module, five individual models (RF, SVM, kNN, MLP, and XGBoost) were tuned, evaluated, and used to build ensemble models using several DES techniques. The best overall ensemble model was then selected based on the average test AUC values over the different numbers of features, and then it was evaluated along with the base models on the test set.

Feature selection algorithms were applied to the training sets in all experiments. The ASVs selected in common by all techniques were kept to obtain a final number of selected features (NSF) between 2 and 15. Different target numbers of features were tested on each method until all desired common values were found.

### 2.3.2 Diagnosing inflammatory bowel disease (IBD)

A first non-curated iteration of the EPheClass pipeline was previously used to diagnose Crohn’s Disease (CD) (Vázquez-González et al., 2023). From this initial version, the pipeline has been improved by adapting the pre-processing of the data to take into account its compositional nature, and by using dynamic ensemble building strategies instead of static ones. Therefore, we additionally applied the new, improved version of EPheClass to the diagnosis of inflammatory bowel disease (IBD), and compared the results obtained with those obtained by other tools employing the same dataset but different classification approaches. For this purpose, we used the Gevers et al. (Gevers et al., 2014) dataset. It contains a total of 1,359 samples, of which 1,023 are from patients with Crohn’s disease (CD), ulcerative colitis (UC), and indeterminate Colitis (IC), and the remaining 336 are from patients without IBD who are considered healthy individuals for the purposes of this study. It contains 16S rRNA gene sequence data belonging to the V4 hypervariable region.

As our pipeline can classify from any type of count table, we used the existing OTU abundance table containing 9,511 OTUs instead of an ASV abundance table. This OTU abundance table can be found in the QIITA database (https://qiita.ucsd.edu/) (Study ID: 1939). A wide range of NSFs were evaluated, from 6 to 38.

### 2.3.3 Classifying antibiotic exposure (DA)

To demonstrate the versatility of the EPheClass pipeline, we applied it to the DIABIMMUNE Antibiotics Cohort (DA) (Yassour et al., 2016) to classify whether a child had been exposed to antibiotics or not, based on OTU profiles derived from stool samples. Microbiome samples were recorded at multiple time points, with sample identifiers encoding the subject and age at the time of collection. OTU data and antibiotic metadata were stored separately and required integration to prepare a classification-ready dataset. A total of 1,101 samples with 3,901 OTUs were used. This dataset is relatively unbalanced and also benefits from data augmentation.

We conducted experiments across feature sets ranging from 2 to 40 OTUs, using the same feature selection and ensemble classification strategy employed in the PD study. CLR transformation was applied to relative abundances, and the most informative OTUs were selected through consensus-based feature selection. Performance was assessed using the same model tuning and evaluation procedure as before, allowing a direct comparison across different phenotypes.

### 2.3.4 Additional experimentation

To strengthen the robustness and generalisability of our findings, we conducted a series of additional experiments on all the datasets we had studied, to assess how sensitive our results were to key methodological choices.

Firstly, to ensure that the results were not biased by the train-test split, we repeated all experiments using five different randomly generated data partitions, each created with a different random seed, while maintaining the proportion of training, validation, and test data.

Next, we studied the effect of feature reduction by evaluating a large number of features, up to 1,500, to assess stability in model performance. Target points were set around 100, 200, 400, 600, 800, 1,000, and 1,500, each with a tolerance of ± 30 features. Our aim was to evaluate the potential loss of sensitivity inherent in any feature selection process, particularly when considering the intersection of the three methods and the impact of imposing a feature cap.

Finally, we evaluated the impact of data augmentation by comparing it with no augmentation and downsampling of the majority class. This study was only applied to those cases where class imbalance was present.

## 3 Results

### 3.1 Periodontal disease (PD) dataset

Table 2 displays the target number of features required to achieve the desired number of ASVs selected by all methods (NSF). Further information on the contribution of each FS method to the final selection of ASVs is provided in Supplementary Table S3.

**TABLE 2:** Target number of features to achieve the desired final number of features (NSF) for each periodontitis subset.

NSF	Target number of features			
	PD_p	PGD_p	PD_s	PGD_s
2	35	20	25	20
3	40	25	30	30
4	46	45	45	50
5	47	47	50	55
6	49	50	55	60
7	50	55	57	-^a^
8	60	65	58	65
9	65	66	59	68
10	71	67	60	70
11	72	70	75	75
12	80	76	80	80
13	85	79	83	83
14	87	80	85	85
15	91	97	90	90

^a^ Could not find consensus of 7 features in common for all three feature selection methods for subset PGD_s.

**FIGURE 4:**
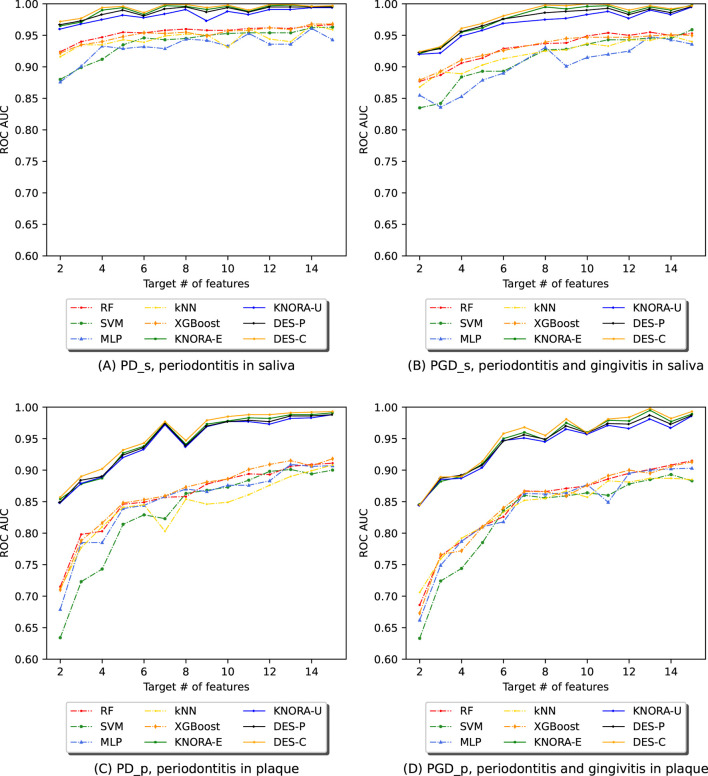
Evolution of the AUC in relation to the number of selected features (NSF) for various models, as evaluated using 10-fold stratified cross-validation on the training data across four different periodontitis subsets: **(A)** periodontitis in saliva, **(B)** periodontitis and gingivitis in saliva, **(C)** periodontitis in plaque, and **(D)** periodontitis and gingivitis in plaque. The following models and ensembles were applied: RF, SVM, MLP, kNN, XGBoost, KNORA-E, KNORA-U, DES-P, and DES-C..

For each experiment, we evaluated the previously listed ML algorithms using the ASV abundance table filtered by the NSFs. We then trained and evaluated various ensembles composed using DES techniques. For each of the four subsets, Figure 4 displays the AUC values obtained by evaluating all base models and ensembles using 10-fold stratified cross-validation on the training set. The Supplementary Material contains comprehensive score tables (Supplementary Table S4–7) of all the evaluated models, one for each plot in Figure 4.

The first row of Figure 4 presents cross-validation AUC values for the saliva samples: one plot for periodontitis alone (PD_s) and another for periodontitis combined with gingivitis (PGD_s). Each plot shows the AUC values of the base models (dashed lines) and the four ensemble methods (solid lines) across different NSFs. Overall, the ensemble models outperform the individual models across all NSFs. Specifically, in Figure 4A (PD_s), all ensembles achieve AUC values above 0.95, while even the lowest-performing individual model, MLP, maintains an AUC above 0.85 for all NSF. Similarly, in Figure 4B (PGD_s), ensemble models again outperform individual models, with their AUC values stabilising after 8 selected features.

The second row of Figure 4 shows cross-validation AUC values for the plaque samples, one for periodontitis alone (PD_p) and the other for periodontitis and gingivitis combined (PGD_p). As in the saliva samples, the ensemble models are the best-performing models for all NSFs in both cases. In Figure 4C, the scores start to drop below 7 features and increase and stabilise above 7, with AUC values over 0.95. Similar results were obtained for subset PGD_p in Figure 4D, where ensembles performed better for larger numbers of features with AUC values of over 0.95.

These comparisons were supported with the paired Venkatraman test (Venkatraman, 1996), evaluating pairwise differences between models for each NSF. As expected, all cases showed significant differences between the ensembles and the individual models. A summary table of these comparisons is provided in the Supplementary Table S13.

To determine the optimal model for each subset, we evaluated the base models and ensembles using real, previously unseen data to form the test set. The AUC values obtained with each model were averaged over the different numbers of features. Table 3 displays the results of the ensembles for all metrics used to evaluate the models, including the F1 score (f1), precision (p), recall (r), accuracy (acc), and the area under the ROC curve (roc_auc). The full version of this table, including the averaged test set AUC values of the base models, can be found in Supplementary Table S8.

**TABLE 3:** Evaluation metrics used for the ensembles: F1 score (f1), precision (p), recall (r), accuracy (acc), and area under the ROC curve (roc_auc). The test set scores of the four periodontitis subsets were averaged over the different numbers of features, ranging from 2 to 15.

Subset	Algorithm	f1	p	r	acc	roc_auc
PD_s	DES-C ensemble	0.885	0.869	0.902	0.908	0.964
	DES-P ensemble	0.891	0.874	0.908	0.913	0.966
	KNORA-E ensemble	0.878	0.871	0.885	0.903	0.964
	KNORA-U ensemble	0.890	0.875	0.907	0.912	0.963
PGD_s	DES-C ensemble	0.867	0.834	0.905	0.891	0.959
	DES-P ensemble	0.871	0.847	0.897	0.896	0.961
	KNORA-E ensemble	0.868	0.841	0.897	0.893	0.959
	KNORA-U ensemble	0.874	0.850	0.900	0.898	0.960
PD_p	DES-C ensemble	0.876	0.876	0.876	0.808	0.834
	DES-P ensemble	0.877	0.885	0.869	0.810	0.838
	KNORA-E ensemble	0.878	0.884	0.873	0.813	0.837
	KNORA-U ensemble	0.875	0.887	0.865	0.810	0.839
PGD_p	DES-C ensemble	0.876	0.892	0.862	0.809	0.823
	DES-P ensemble	0.875	0.903	0.849	0.809	0.824
	KNORA-E ensemble	0.877	0.895	0.861	0.810	0.819
	KNORA-U ensemble	0.874	0.905	0.847	0.808	0.825

Based on the results of the test set, the ensemble techniques demonstrated highly similar performance, with a maximum difference of just 0.06 in the AUC values. These comparisons were supported by the Venkatraman paired test (Venkatraman, 1996). Evaluating pairwise differences between models for each NSF, only a small proportion of model comparisons showed statistically significant differences, averaging around 20%. This means that, out of all 1,660 model comparisons performed across different NSFs and subsets in the test set, only about 20% indicated statistically significant differences. A summary table of these comparisons is provided in the Supplementary Table S9.

Given these minor differences, DES-P was selected as the reference ensemble method, as it consistently achieved the highest AUC values across most subsets. For PD_s, DES-P obtained an F1 score of 0.891, precision of 0.874, recall of 0.908, accuracy of 0.913, and an AUC of 0.966. For PGD_s, DES-P achieved an F1 score of 0.871, precision of 0.847, recall of 0.897, accuracy of 0.896, and AUC of 0.961. For PD_p, DES-P recorded an F1 score of 0.877, precision of 0.885, recall of 0.869, accuracy of 0.810, and AUC of 0.838. Finally, for PGD_p, DES-P reached an F1 score of 0.875, precision of 0.903, recall of 0.849, accuracy of 0.809, and AUC of 0.824.

**FIGURE 5:**
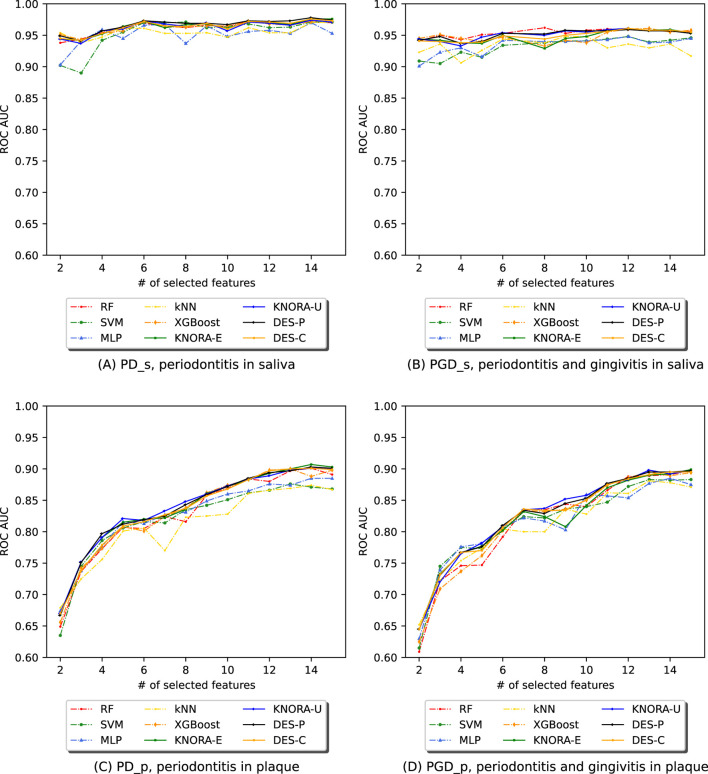
Evolution of the AUC in relation to the number of selected features (NSF) for model comparison across four different periodontitis subsets: **(A)** periodontitis in saliva, **(B)** periodontitis and gingivitis in saliva, **(C)** periodontitis in plaque, and **(D)** periodontitis and gingivitis in plaque. Results for RF, SVM, MLP, kNN, XGBoost, and DES-P for each subset were obtained using the test set data.

In Figure 5, the test set AUC values for the base techniques and ensembles are presented. The first row displays two plots for the saliva samples, one for periodontitis alone (PD_s) and the other for periodontitis and gingivitis (PGD_s). The Supplementary Material contains comprehensive score tables (Supplementary Table S10–13) of all the evaluated models, one for each plot in Figure 5.

Figure 5A (PD_s) and Figure 5B (PGD_s) show that, while ensembles and individual models achieved similar AUC values, the ensemble presented greater stability across the range of selected features and maintained higher AUC values even with fewer features. The second row of Figure 5 presents two plots for the plaque samples: one for periodontitis alone (PD_p) and the other for periodontitis combined with gingivitis (PGD_p). As seen for saliva, ensembles and individual models achieved similar AUC values, but the ensemble presented greater stability across the different NSFs. However, in both plaque subsets, the scores begin to drop below 8 features, but remain stable and slightly increase above this value.

Overall, the results indicate that the ensembles performed considerably better in cross-validation results (training set) and were more stable than the individual models with the test set. Notably, the periodontitis samples alone performed similarly to the combination of periodontitis and gingivitis samples. As for the plaque subsets, which were twice the size of the saliva subsets, the ensembles tended to enhance the results for a larger number of features. In this case, the samples of periodontitis alone also showed similar results to the samples of both periodontitis and gingivitis combined.

Table 4 presents the analysis of the performance of various feature numbers on the test set using the reference ensemble, DES-P, for all subsets. The three best-performing models were selected for each subset based on the AUC values.

**TABLE 4:** F1 score (f1), precision (p), recall (r), accuracy (acc), and area under the ROC curve (roc_auc) for the reference ensemble, and its performance with different numbers of features with the test set for each periodontitis subset. Only the three best number of features are shown, ordered per subset from lowest to highest NSF.

Subset	Algorithm	NSF	f1	p	r	acc	roc_auc
PD_s	DES-P	13	0.913	0.881	0.947	0.929	0.973
		14	0.903	0.913	0.894	0.925	0.978
		15	0.898	0.903	0.894	0.921	0.974
PGD_s	DES-P	9	0.854	0.837	0.872	0.883	0.958
		12	0.873	0.835	0.915	0.895	0.959
		14	0.867	0.833	0.904	0.891	0.957
PD_p	DES-P	13	0.903	0.918	0.889	0.852	0.897
		14	0.913	0.924	0.902	0.867	0.903
		15	0.904	0.909	0.899	0.852	0.901
PGD_p	DES-P	13	0.899	0.927	0.873	0.846	0.895
		14	0.907	0.933	0.882	0.857	0.895
		15	0.913	0.929	0.897	0.865	0.897

We can observe that there is a minimal variation in the AUC between the three best, with differences of only up to 0.006, indicating great consistency and stability in classification performance. Thus, we decided to select the best model based on the NSF, prioritising small numbers of features. As such, the DES-P ensemble achieved the best performance using 13 features for PD_s, 9 features for PGD_s, 13 features for PGD_p, and 13 features for PD_p.

As outlined in Section 2.3.4, to further evaluate the results, these experiments were repeated for five different data partitions. In most cases, the unpaired Venkatraman test (Venkatraman, 2000) showed no statistically significant differences between the partitions. This indicates that performance was generally consistent regardless of the specific train-test split. Specifically, when evaluating the test sets, the different partitions within each subset yielded highly consistent results, with similarity across seeds exceeding 90%. More information on Supplementary Table S14.

Additionally, we evaluated the impact of data augmentation. The results obtained in the plaque subsets with augmentation were compared to those obtained with no augmentation and with downsampling of the majority class. The unpaired Venkatraman test revealed no statistically significant differences among the three strategies. This suggests that the models can generalise effectively even when trained on unbalanced data, likely due to the inherent distinctiveness of the classes. However, data augmentation is still standard practice for addressing class imbalance in predictive modelling. As it does not negatively impact performance, its use is recommended to ensure methodological rigour (Kuhn and Johnson, 2013). More information on Supplementary Table S15, 16.

Furthermore, we evaluated larger numbers of features ranging from 100 to 1,500 to assess stability in model performance. We observed that, for over 100 features, the ROC AUC stabilises for both the cross-validation and test results. The plots corresponding to these larger feature numbers are available in the Supplementary Figures S1, 2.

Lastly, it is of interest to analyse the specific ASVs that were involved in the classification. To accomplish this, the taxonomy of each ASV was obtained and compared among the four subsets. Table 5 illustrates that certain features are duplicated between the saliva subsets (with and without gingivitis), as well as for the plaque subsets. Additionally, it was found that feature ASV00242 is significant for PD_s, PGD_s, and PGD_p, which corresponds to the bacterial species Bacteroidaceae [G-1], bacterium_HMT272. The study’s findings indicate that the genus *Streptococcus* is present in all subsets.

**TABLE 5:** ASVs used in the best model for each periodontitis subset, including their taxonomy.

	PD_s	PGD_s	PD_p	PGD_p	Genus	Species
ASV02511	✓				Actinomyces	sp.HMT172
ASV01745	✓				Prevotella	melaninogenica
ASV00242	✓	✓		✓	Bacteroidaceae [G-1]	bacterium_HMT272
ASV00195	✓	✓			Treponema	unclassified
ASV00121	✓	✓			Streptococcus	unclassified
ASV00085	✓				Streptococcus	unclassified
ASV00027	✓	✓			Streptococcus	vestibularis
ASV00015	✓	✓			Tannerella	forsythia
ASV00006	✓				Rothia	aeria
ASV00005	✓				Veillonella	dispar
ASV00564	✓				Haemophilus	sputorum
ASV00643	✓	✓			Actinomyces	sp.HMT169
ASV00155	✓				Streptococcus	unclassified
ASV01554		✓			Veillonella	rogosae
ASV00036		✓			unclassified Saccharibacteria (TM7) [F-1]	unclassified
ASV00559		✓			Butyrivibrio	sp.HMT080
ASV01577			✓	✓	Cardiobacterium	hominis
ASV01534				✓	Streptococcus	oralis_subsp.dentisani_clade_058
ASV01025				✓	Lactobacillus	ultunensis
ASV00929				✓	Porphyromonas	catoniae
ASV00721				✓	Cutibacterium	acnes
ASV00650			✓	✓	unclassified Saccharibacteria (TM7) [F-1]	unclassified
ASV00380			✓	✓	Streptococcus	unclassified
ASV00233				✓	Haemophilus	sp.HMT036
ASV00206				✓	Actinomyces	massiliensis
ASV00189			✓	✓	Peptostreptococcaceae [XI][G-6]	nodatum
ASV00108			✓	✓	Pseudomonas	fluorescens
ASV00030				✓	Fusobacterium	unclassified
ASV01669			✓		Leptotrichia	wadei
ASV01356			✓		Rothia	dentocariosa
ASV01320			✓		Veillonella	unclassified
ASV01234			✓		Cupriavidus	gilardii
ASV00793			✓		Peptostreptococcaceae [XI][G-6]	minutum
ASV00213			✓		Mycoplasma	faucium
ASV01679			✓		Veillonella	unclassified
ASV00199			✓		Corynebacterium	durum

### 3.2 Inflammatory bowel disease (IBD) dataset

For IBD, Supplementary Table S17 shows the target number of features required to achieve the desired number of OTUs selected by all methods (NSF).

**FIGURE 6:**
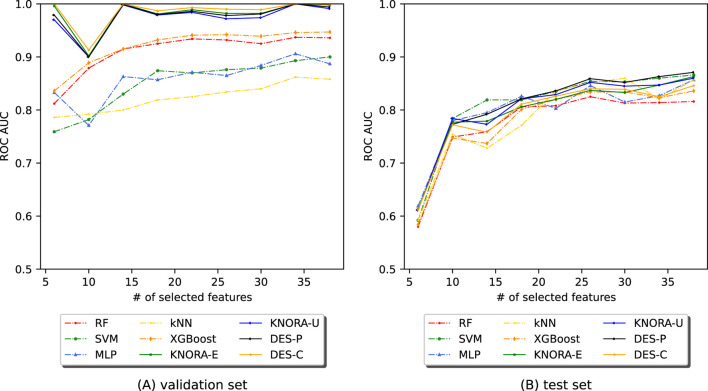
Evolution of the AUC as a function of the number of selected features (NSF) for different models applied to the IBD dataset. The models were analysed using **(A)** cross-validation and **(B)** the test set. The models and ensembles used were RF, SVM, MLP, kNN, XGBoost, KNORA-E, KNORA-U, DES-P, and DES-C.

Figure 6 shows the AUC values after applying EPheClass to the IBD dataset. The Supplementary Material contains comprehensive score tables (Supplementary Tables S18, 19) of all the evaluated models, one for each plot in Figure 6. Specifically, Figure 6A shows that the ensembles perform significantly better than the base models with cross-validation, while performing similarly to each other. Figure 6B shows that the ensembles and base models perform comparably on the test set, performing worse with fewer numbers of features, but more stable and slightly better for larger numbers.

These comparisons were also supported by the paired Venkatraman test (Venkatraman, 1996). As expected, cross-validation revealed significant differences between the ensembles and the individual models. With the test set, however, only a small proportion of 22% of the 300 model comparisons across different NSFs showed statistically significant differences. A summary table of these comparisons is provided in the Supplementary Table S20.

Table 6 displays the averaged test set results of the ensembles for all metrics used to evaluate the models. The AUC values obtained with each model have been averaged over the different numbers of features. The full version of this table, including the averaged test set AUC values of the base models, can be found in Supplementary Table S21. It shows that the ensembles perform similarly to each other in terms of AUC value, with a difference of only 0.017. Due to the lack of significant differences, we selected DES-P as the reference ensemble, as it had the highest AUC on average. The DES-P ensemble achieved an F1 score of 0.854, a precision of 0.865, a recall of 0.844, an accuracy of 0.783, and an AUC of 0.809.

**TABLE 6:** Evaluation metrics used for the ensembles: F1 score (f1), precision (p), recall (r), accuracy (acc), and area under the ROC curve (roc_auc). The test set scores of the IBD dataset were averaged over different numbers of features, ranging from 6 to 38.

Dataset	Algorithm	f1	p	r	acc	roc_auc
IBD	DES-C ensemble	0.857	0.835	0.880	0.778	0.792
	DES-P ensemble	0.854	0.865	0.844	0.783	0.809
	KNORA-E ensemble	0.855	0.850	0.860	0.780	0.797
	KNORA-U ensemble	0.859	0.869	0.850	0.790	0.802

Table 7 shows the results of analysing the performance of different numbers of features on the test set using the selected ensemble, DES-P. As there was very little variation in the AUC between the three best models, regardless of the NSF, the model with the fewest features was chosen. Consequently, the DES-P ensemble performed best with just 26 features.

**TABLE 7:** F1 score (f1), precision (p), recall (r), accuracy (acc), and area under the ROC curve (roc_auc) for the best algorithm on average, DES-P, and its performance with different numbers of features on the test set for the IBD dataset. Only the three best number of features are shown, with a small number ordered from lowest to highest NSF.

Dataset	NSF	f1	p	r	acc	roc_auc
IBD	26	0.866	0.890	0.844	0.804	0.859
	34	0.885	0.883	0.886	0.826	0.863
	38	0.884	0.899	0.870	0.828	0.871

The additional experiments confirmed the same conclusions as in the PD analysis, showing consistent performance with no statistically significant differences for different data partitions (Supplementary Table S22) and for different class imbalance management strategies (Supplementary Table S23).

As with the PD dataset, we evaluated larger numbers of features from 100 to 1,500 to assess stability in model performance. We observed that, for over 100 features, the ROC AUC stabilises for both the cross-validation and test results. The plots corresponding to these larger feature numbers are available in the Supplementary Figure S3.

### 3.3 Antibiotics exposure (DA) dataset

**FIGURE 7:**
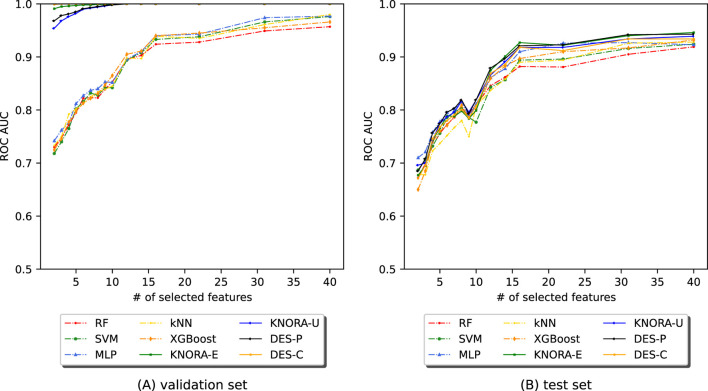
Evolution of the AUC as a function of the number of selected features (NSF) for different models applied to the DA dataset. Models were analysed using **(A)** cross-validation and **(B)** the test set. Models included RF, SVM, MLP, kNN, XGBoost, KNORA-E, KNORA-U, DES-P, and DES-C.

Figure 7 shows the AUC values obtained by applying EPheClass to the antibiotics exposure dataset. As with the other cohorts, we evaluated model performance across different numbers of selected features (NSF), ranging from 2 to 40. Detailed score tables for each evaluated model are provided in Supplementary Table S24, 25.

Figure 7A shows that the ensemble models consistently outperform the base classifiers under cross-validation. In contrast, Figure 7B shows that performance on the test set is more variable, especially for very small feature sets. However, performance stabilises with more features. The paired Venkatraman test (Venkatraman, 1996) revealed statistically significant differences between the ensembles and individual models in nearly all cross-validation cases. On the test set, however, only a minority of comparisons showed significant differences.

Table 8 reports the averaged test performance for the ensemble models across all tested feature sizes. DES-P achieved the highest average AUC and was thus selected as the reference model for further analysis. The full version of this table, including the averaged test set AUC values of the base models, can be found in Supplementary Table S26.

**TABLE 8:** Evaluation metrics used for the ensembles: F1 score (f1), precision (p), recall (r), accuracy (acc), and area under the ROC curve (roc_auc). The test set scores of the DA dataset were averaged over different numbers of features, ranging from 2 to 15.

Dataset	Algorithm	f1	p	r	acc	roc_auc
DA	DES-C ensemble	0.686	0.684	0.690	0.756	0.820
	DES-P ensemble	0.683	0.694	0.673	0.760	0.831
	KNORA-E ensemble	0.687	0.684	0.692	0.757	0.823
	KNORA-U ensemble	0.694	0.699	0.689	0.766	0.826

Table 9 summarises the top three performing feature counts on the test set using the DES-P ensemble. As there was very little variation in the AUC between the three best models, regardless of the NSF, the model with the fewest features was chosen. Consequently, the model performed best with just 22 features, demonstrating the effectiveness of dimensionality reduction.

**TABLE 9:** F1 score (f1), precision (p), recall (r), accuracy (acc), and area under the ROC curve (roc_auc) for the best algorithm on average, DES-P, and its performance with different numbers of features on the test set for the DA dataset. Only the three best number of features are shown, ordered from lowest to highest NSF.

Dataset	NSF	f1	p	r	acc	roc_auc
DA	22	0.814	0.817	0.811	0.858	0.923
	31	0.865	0.872	0.858	0.897	0.942
	40	0.858	0.858	0.858	0.891	0.943

As in the other datasets, we also explored larger feature sizes (from 100 to 1,500) and found that both cross-validation and test AUC stabilised beyond approximately 100 features. Full plots are available in the Supplementary Figure S4.

The main goal for the antibiotic exposure dataset was to demonstrate the versatility of the pipeline. Therefore, no additional analyses on data partitions or the impact of augmentation were performed, as the results obtained with previous evaluations provided enough information.

## 4 Discussion and conclusion

This work proposes a curated pipeline for classifying phenotypes with 16S rRNA gene sequenced samples. The pipeline promotes reproducibility and reduces computational costs through feature selection. The ensemble models achieved the best results for all datasets and subsets, based on a trade-off between high scores and low target number of features, as shown in Figure 4.

This evaluation is based solely on objective data, using the AUC value plots, the paired and unpaired Venkatraman tests, and the comparison of the multiple performance metrics.

### 4.1 Periodontal disease (PD) dataset

Several works in the literature have addressed the prediction of periodontal disease in the oral cavity. However, as shown in Table 10, they have not followed a rigorous procedure to perform a reliable and accurate prediction. Failure to meet the basic requirements for building adequate predictive models, including sufficiently large sample size, data set splitting and cross-validation, and an adequate number of features, means that the results cannot be considered valid (Kuhn and Johnson, 2013), making them unreliable and incomparable to ours.

**TABLE 10:** Comparison of EPheClass with other works diagnosing periodontal disease. P, periodontitis; H, healthy; NSF, number of selected features; DS, Data split; CV, cross-validation.

Work	Collection site	Dataset size	NSF	DS and CV	f1	p	r	acc	roc_auc
Lundmark et al. (2019)	Saliva	66P - 48H	Unspecified	No	-	-	-	79.52%	-
Narita and Kodama (2022)	Saliva	12P - 41H	Unspecified	No	72.7%	80%	67%	88.7%	-
Na et al. (2020)	Supragingival	210P - 62H	Unspecified	Only CV	90.5%	-	-	85.3%	-
Chen et al. (2021) ^a^	Subgingival	123P - 96H	Unspecified	Only DS	-	-	-	-	91.8%
EPheClass (ours)	Saliva	314P - 483H	13	Yes	91.3%	88.1%	94.7%	92.9%	97.3%
	Plaque^b^	1298P - 369H^c^	13	Yes	90.3%	91.8%	88.9%	85.2%	89.7%

^a^ Model specific for supragingival periodontitis, not generalizable.

^b^ Subgingival + supragingival samples.

^c^ Size of the original dataset. An additional 630 augmented healthy samples were added to the training set.

When constructing predictive models, it is crucial to use sufficiently large and balanced datasets to enable proper learning and generalisation. Table 10 shows that the works mentioned either used highly unbalanced data (Narita and Kodama, 2022; Na et al., 2020) or datasets that were not large enough (Lundmark et al., 2019; Narita and Kodama, 2022; Chen et al., 2021). EPheClass utilises considerably larger datasets (314P - 483H for saliva and 1298P - 999H for plaque) and even performs data augmentation to ensure class balance.

The relevance of the number of features used should not be overlooked, as an excessive amount can result in overly complex models. None of the studies provided a clear indication of the number of features used (Lundmark et al., 2019; Narita and Kodama, 2022; Chen et al., 2021; Na et al., 2020). However, Na et al. (2020) reported that they performed feature selection, which resulted in improved outcomes. In contrast, EPheClass employs between 2 and 15 features, with the optimal score achieved using 13 features for saliva and 13 for plaque.

To ensure reliable results and avoid overfitting, models must undergo evaluation through cross-validation and the use of real, unseen data. Neither Lundmark et al. (2019) nor Narita and Kodama (2022) employed cross-validation or divided the dataset into training and testing sets. Na et al. (2020) exclusively employed cross-validation, while Chen et al. (2021) utilised only one training set and one test set. In contrast, EPheClass performs both data splitting and cross-validation. To make valid comparisons between studies, it is important to ensure that they meet these minimum criteria.

The studies achieved high scores, with Lundmark et al. (2019) obtaining an accuracy of 79.52 % , Narita and Kodama (2022) with a higher accuracy of 88.7 % , a recall of 67 % , precision of 80 % and an F1 score of 72.7 % , Na et al. (2020) with a high F1 score of 90.5 % and an accuracy of 85.3 % , and Chen et al. (2021) with a high AUC of 91.8 % . However, as these studies do not meet the basic criteria mentioned earlier, their scores cannot be directly compared to ours.

Furthermore, Chen et al. (2021) achieved a higher AUC by using a periodontitis-specific index on subgingival samples, which is not applicable to other pathologies and mixes features from different regions of the 16S rRNA gene. In contrast, EPheClass can predict any polymicrobial pathology. Our pipeline meets the basic conditions and achieves with DES-P for the PD dataset an average test AUC of 0.931, an F1 score of 0.892, a precision of 0.891, a recall of 0.895, and an accuracy of 0.903, using only 9 to 13 selected features.

In our PD dataset (Figures 4, 5), saliva subsets with and without gingivitis (PGD_s vs PD_s) performed similarly, as did plaque subsets (PGD_p vs PD_p). Although gingivitis, which is recognised as an early stage of periodontal involvement, adds heterogeneity and classification difficulty, models generalise well across all subsets. Moreover, differences in overall performance can be noted depending on the site of sample collection. Saliva samples yielded better results than plaque, especially with fewer features (Figures 4, 5). Specifically, DES-P reached an AUC of 97.3 % in saliva samples and 89.7 % in plaque samples, using only 13 features.

Therefore, it appears that saliva samples contain sufficient information to accurately classify periodontitis. Furthermore, satisfactory results can still be attained even when gingivitis is present. All subsets demonstrate a high degree of predictive capacity with few features, highlighting the positive impact of feature selection.

Given that all ensembles performed similarly according to the Venkatraman test, DES-P was selected as the representative ensemble, as it achieved the highest average test score across feature numbers. Table 3 showed that ensembles were the best models for all PD subsets. Table 4 confirmed that the DES-P model performed strongly and stably on the test data, achieving peak AUC values ranging from 0.895 to 0.973, depending on the subset and the number of features.

### 4.2 Inflammatory bowel disease (IBD) dataset

We also evaluated the pipeline on an IBD dataset, where the DES-P ensemble demonstrated strong predictive performance on the test set while using only 26 features. These results are better than those obtained by other studies using the same dataset to classify this disease using different approaches, as shown in Table 11. Asgari et al., 2018 proposed Micropheno to classify phenotypes using k-mer abundance tables and RF models. Unal et al. (2023) evaluated different machine learning algorithms, including RF, SVM, and kNN, to predict diseases using k-mer abundance tables. Zhao et al. (2021) proposed Read2Pheno, a phenotype classifier that uses Convolutional Neural Networks (CNN), Recurrent Neural Networks (RNN), and attention mechanisms to individually classify each sequence in the samples. As a final comparison, Syama et al. (2023) built graphs, considering each metagenomic sample as a node in the graph and capturing the relationship between the samples using a proximity measure, and used them as input for a boosting GraphSAGE model that predicts the status of a sample as sick or healthy.

**TABLE 11:** Comparison of EPheClass with other works for the diagnosis of IBD. D: IBD. H, healthy; NSF, number of selected features; DS, data split; CV, cross-validation.

Work	Dataset size	NSF	DS and CV	f1	p	r	acc	roc_auc
Asgari et al. (2018) ^a^	731D - 628H	4,096	Only CV	76 %	76 %	76 %	-	-
Unal et al. (2023)	1,023D - 336H	4,096	Only DS	76.5 %	76.6 %	-	76.5 %	82.1 %
Zhao et al. (2021)	210D - 210H	Unspecified	Yes	-	-	-	83.3 %	-
Syama et al. (2023)	1,023D - 336H	1,250	Only DS	95 %	-	-	95 %	93 %
EPheClass (ours)	1,023D - 336H^b^	26	Yes	86.6 %	89.0 %	84.4 %	80.4 %	85.9 %

^a^ Scores obtained after cross-validation, in contrast to all other rows showing the test set results.

^b^ Size of the original dataset. An additional 480 augmented healthy samples were added to the training set.

These tools proposed in the literature achieve results that, in some cases, seem comparable to or even surpass our own. However, we consider ours to be better because, unlike our proposal, these tools use thousands of features. The use of parsimonious models with few features is crucial from a clinical point of view (Steyerberg, 2009), as simpler models facilitate the interpretability of the results, avoid overfitting, and increase the clinical applicability. In general, overly complex models should be avoided (Moons et al., 2015). In addition, they do not follow some of the key steps described earlier in this section to achieve a reliable prediction.

In more detail, Asgari et al., 2018 used 4,096 features, corresponding to 6-mers, and only seem to evaluate the classifier with cross-validation, while we provide both the cross-validation and test set results. Our cross-validation results outperform theirs. Unal et al. (2023) also used 4,096 features, did not consider class imbalance, and only performed data splitting, without showing the cross-validation results. Zhao et al. only used 442 out of the 1,359 samples for training and testing, which reduces the diversity and variability of the data. Moreover, the only metric shown is the accuracy. Finally, Syama et al. (2023) do not seem to perform any cross-validation, hyperparameter tuning or feature selection. While they use 1,250 features, we achieve highly promising scores with only 26 features. Furthermore, the F1 scores of the traditional models evaluated in their study show the neglected class imbalance (e.g., F1 score of 0.57 with RF), which is recognised and not taken into account in the evaluation. They achieve higher scores than our proposal, but with an increased number of features and without taking into account the strong class imbalance. We decided to evaluate our proposal under their conditions to see if such higher scores could be achieved. Without balanced classes, cross-validation, hyperparameter tuning, and feature selection techniques, we achieved even better results than those shown by Syama et al. (2023). The DES-P ensemble scored 1 on all metrics, both on the test set and the training set. Nevertheless, the criteria already mentioned for reliable and accurate classifiers should be followed in order to avoid overfitting and to achieve proper learning and generalisation.

### 4.3 Antibiotic exposure (DA) dataset

Unlike the PD and IBD datasets, the DA dataset was not included with the aim of comparing our results against existing studies addressing the same classification task. Instead, it was used mainly for benchmarking purposes, allowing us to test and compare our pipeline against other existing phenotype classifiers. This dataset served to further demonstrate the pipeline’s versatility in handling different prediction problems.

### 4.4 Benchmarking

Additionally, we have benchmarked other phenotype classifier tools to compare their performance with our pipeline using the test sets of the PD, IBD, and DA datasets (see Table 12). These tools are MegaD (Mreyoud et al., 2022), which employs convolutional neural networks to classify microbiome data, and DeepMicro (Oh and Zhang, 2020), which applies autoencoder-based representation learning. For MegaD, we used the default experimental configuration provided. In the case of DeepMicro, although it was designed to perform feature reduction via autoencoders, we were unable to run the pertinent code. Therefore, we evaluated only the classifier (SVM), which still performed well in their results.

**TABLE 12:** Performance benchmark of EPheClass using DES-P compared to existing methods for PD and IBD diagnosis, and for antibiotic exposure detection. NSF, number of selected features; DS, data split; CV, cross-validation.

Dataset	Work	NSF	DS and CV	f1	p	r	acc	roc_auc
PD	Mreyoud et al. (2022)	10,577	Only DS	90.5 %	89.5 %	91.4 %	92.5 %	96.7 %
	Oh and Zhang (2020)	10,577	Yes	81.2 %	81.7 %	80.8 %	85.13 %	87.1 %
	EPheClass (ours)	13	Yes	91.3 %	88.1 %	94.7 %	92.9 %	97.3 %
IBD	Mreyoud et al. (2022)	9,511	Only DS	85.5 %	80.1 %	90.8 %	76.9 %	72 %
	Oh and Zhang (2020)	9,511	Yes	81.8 %	84.4 %	79.4 %	73.5 %	71.5 %
	EPheClass (ours)	26	Yes	86.6 %	89 %	84.4 %	80.4 %	85.9 %
DA	Mreyoud et al. (2022)	3,901	Only DS	77.5 %	91.5 %	67.2 %	84.5 %	88.6 %
	Oh and Zhang (2020)	3,901	Yes	82.2 %	85.9 %	78.8 %	86.8 %	92.4 %
	EPheClass (ours)	22	Yes	81.4 %	81.7 %	81.1 %	85.8 %	92.3 %

While all methods produced competitive results, EPheClass stands out by achieving comparable or superior performance while using dramatically fewer features, fewer than 30 in all cases, compared to the thousands used by other tools. This emphasises the parsimony and interpretability of our pipeline, making it especially suitable for clinical settings.

To the best of our knowledge, no other polymicrobial disease classification pipeline has achieved such promising results with only a few features. This is due to the combination of ASV abundance filtering and Recursive Feature Elimination (RFE) to reduce data dimensionality, as well as the use of dynamic ensemble models for classification.

### 4.5 Generalisation and limitations of the pipeline

We have proven that the pipeline performs well in classifying different phenotypes, i.e., periodontal disease, IBD, and antibiotic exposure. This robustness was further supported by additional experiments using multiple data partitions, extended feature sets of up to 1,500 features, and different class imbalance management strategies (augmentation, no augmentation, and downsampling). This highlights the generalisability of the approach to other study niches and different sample types.

However, our pipeline’s ability to accurately classify phenotypes, as well as any other classifier from abundance tables, can be influenced by the sequencing depth of the data. Each training sample must include a minimum number of sequences, which depends on the sample type and the quality of the sequencing process. A greater depth increases the ability to distinguish between classes.

In conclusion, the proposed pipeline achieved a balance between high scores and a smaller number of features through feature selection and dynamic ensembles. Additionally, it ensures reproducibility of results and reduces computational costs by significantly reducing data dimensionality. The results are now more comprehensible, allowing for the identification of specific bacteria that may cause or influence the disease under study. Moreover, our findings exceed those of prior studies that did not employ a rigorous methodology. We are convinced that this pipeline is capable of accurately classifying any heterogeneous polymicrobial disease dataset and producing reliable and representative results.

## Data Availability

The PD used and analysed in this study was compiled by our research team. The tables can be found in https://github.com/Oral-Sciences-Research-Group/Epheclass_dataset. The IBD dataset was obtained from the Gevers et al. (2014) study. The OTU abundance table can be found in the QIITA database (https://qiita.ucsd.edu/) (Study ID: 1939). The DA dataset was obtained from the Yassour et al. (2016) study. The OTU abundance table can be found in https://diabimmune.broadinstitute.org/diabimmune/antibiotics-cohort. The pipeline code is available at https://gitlab.citius.usc.es/lara.vazquez/epheclass.
