# Supplementary material for: EPheClass: ensemble-based phenotype classifier from 16S rRNA gene sequences
Source: Front Bioinform. 2025 Sep 30;5:1514880. doi: 10.3389/fbinf.2025.1514880 (PMC12518240; doi:10.3389/fbinf.2025.1514880)
Supplement: Supplementary file 5 [file DataSheet1.pdf]

Supplementary Material

1 SUPPLEMENTARY INFORMATION: PERIODONTITIS DATA PREPROCESSING

1.1 Periodontitis data download

Raw sequences in fastq format were downloaded from the SRA databases using a Bash script in conjunction with the SRA Toolkit software (Leinonen et al. (2010)). For the remaining databases, sequences were downloaded manually. Only files containing paired-end sequences with lengths of 2x250 or 2x300 bp were accepted for download, or alternatively, sequences that had already been merged with a length greater than 350 bp. All sequences are from the V3-V4 variable region and were sequenced using the recommended Illumina forward and reverse primers or primers with a maximum of four different positions compared to the Illumina primers, which are the following:

V3-V4-Forward (5'-CCTACGGGNGGCWGCAG-3)

V3-V4-Reverse (5'-GACTACHVGGGTATCTAATCC-3)

1.2 Preprocessing and quality control of sequences

The samples undergo a quality control process using USEARCH (Edgar (2010)). The 2x250 bp sequences were aligned and merging into a single contig, allowing a maximum of 5 mismatches with 90% similarity in the overlapping region of both sequences (forward and reverse). For the 2x300 bp sequences, a maximum of 10 mismatches and 80% similarity were allowed. In both cases, a minimum overlap of 20 bp was required. Merged sequences with an expected maximum error greater than 1 or a length less than 300 bp were discarded. As a large number of samples were processed, quality control was performed individually for each sample. Once all downloaded samples were preprocessed and quality control was completed, a Bash script was used to merge all sequences into a single file containing unique sequence and sample identifiers.

Next, the ASV pipeline proposed by *mothur* (Schloss et al. (2009)) was applied with slight modification. Sequences with more than 8 homopolymers or chimeras were discarded. Unique sequences were taxonomically assigned using the Bayesian algorithm (Wang et al. (2007)) implemented in *mothur* against an oral sequence database proposed by Escapa et al. (2020). Two tables were created: one with the count of samples and taxa, and another with the taxonomic hierarchy up to the ASV level for each identified taxon. Finally, we exported the count table for further analysis in Python. A total of 10,577 ASVs were identified, all of which have the potential to be predictors.

REFERENCES

Leinonen R, Sugawara H, Shumway M. The sequence read archive. *Nucleic Acids Research* **39** (2010) D19–D21. doi:10.1093/nar/gkq1019.

Edgar RC. Search and clustering orders of magnitude faster than BLAST. *Bioinformatics* **26** (2010) 2460–2461. doi:10.1093/bioinformatics/btq461.

Schloss PD, Westcott SL, Ryabin T, Hall JR, Hartmann M, Hollister EB, et al. Introducing mothur: open-source, platform-independent, community-supported software for describing and comparing microbial

communities. *Applied and Environmental Microbiology* **75** (2009) 7537–7541. doi:10.1128/AEM.01541-09.

Wang Q, Garrity GM, Tiedje JM, Cole JR. Naïve bayesian classifier for rapid assignment of rRNA sequences into the new bacterial taxonomy. *Applied and Environmental Microbiology* **73** (2007) 5261–5267. doi:10.1128/aem.00062-07.

Escapa IF, Huang Y, Chen T, Lin M, Kokaras A, Dewhirst FE, et al. Construction of habitat-specific training sets to achieve species-level assignment in 16s rRNA gene datasets. *Microbiome* **8** (2020). doi:10.1186/s40168-020-00841-w.

2 SUPPLEMENTARY TABLES AND FIGURES

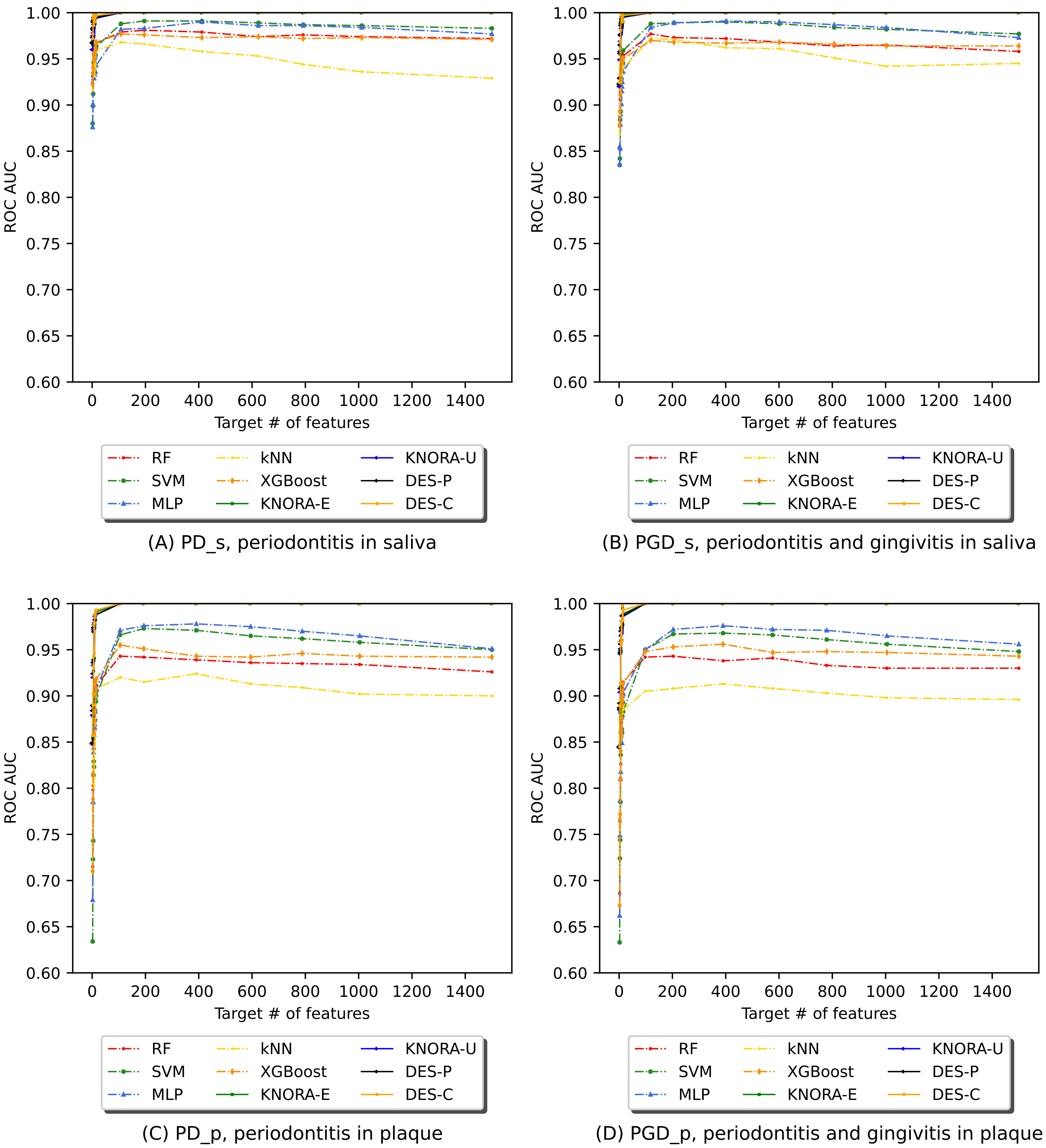

Figure S1. Evolution of the AUC in relation to a larger number of selected features (NSF) for various models applied to four different periodontitis subsets. The models were analysed using cross-validation. The following models and ensembles were applied: RF, SVM, MLP, kNN, XGBoost, KNORA-E, KNORA-U, DES-P and DES-C.

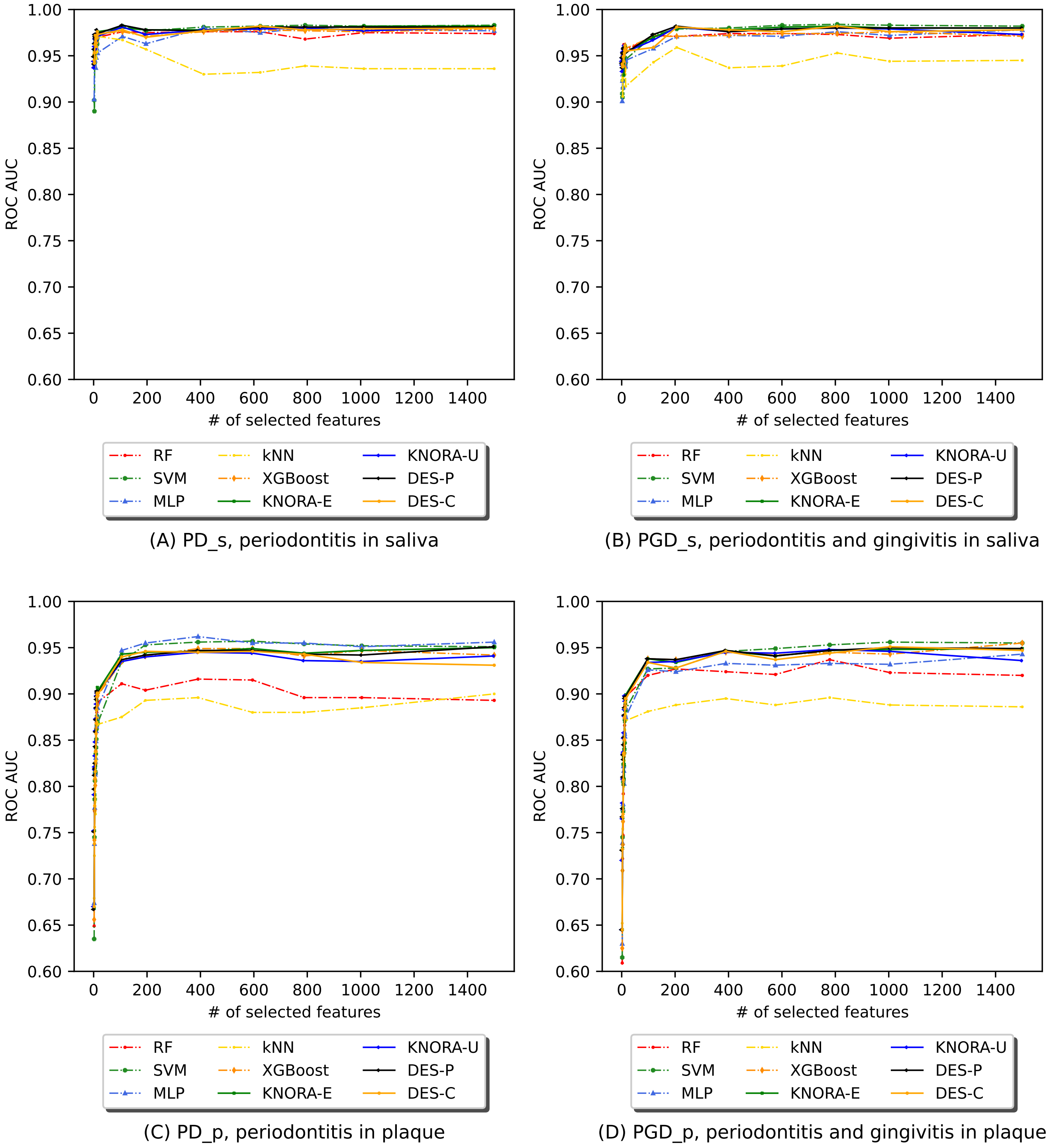

Figure S2. Evolution of the AUC in relation to a larger number of selected features (NSF) for model comparison. Results for RF, SVM, MLP, kNN, XGBoost, KNORA-E, KNORA-U, DES-P and DES-C for each subset, were obtained using the test set data.

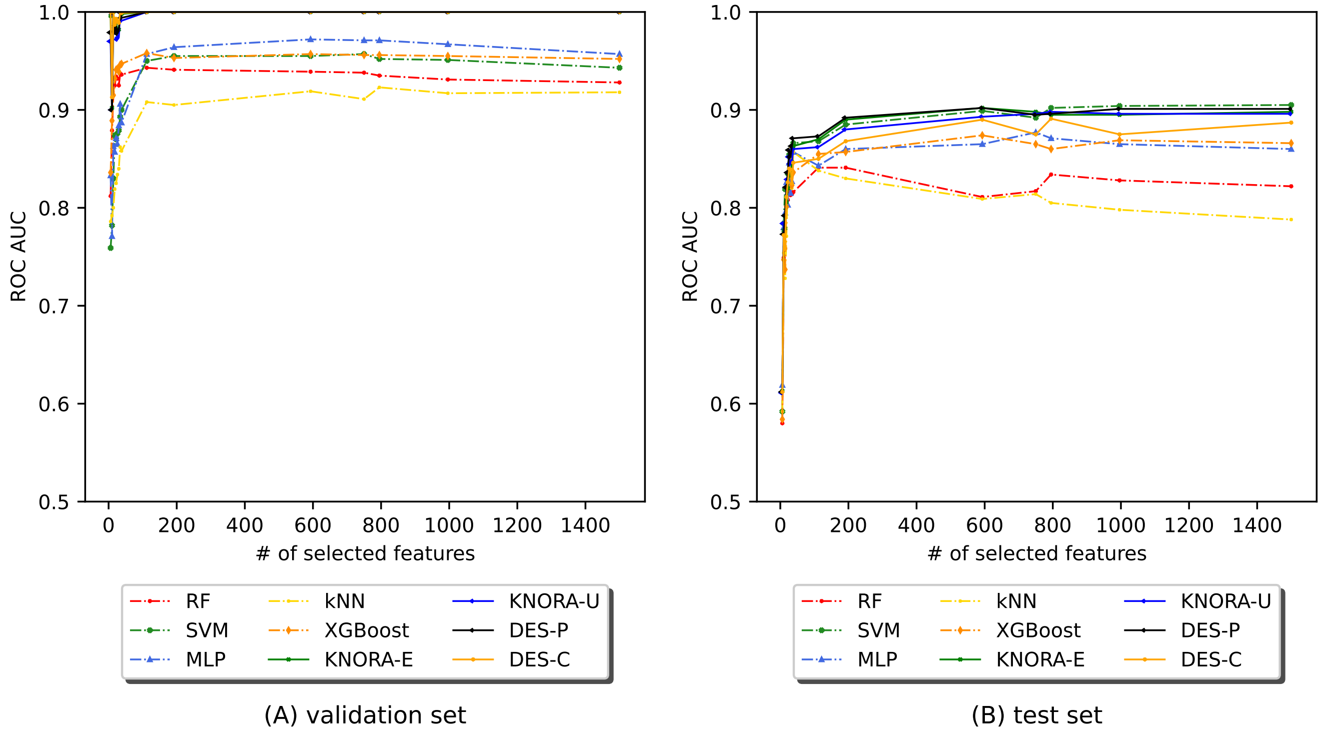

Figure S3. Evolution of the AUC as a function a larger number of selected features (NSF) for different models applied to the IBD dataset. The models were analysed using (A) cross-validation and, (B) the test set. The following models and ensembles used were RF, SVM, MLP, kNN, XGBoost, KNORA-E, KNORA-U, DES-P and DES-C.

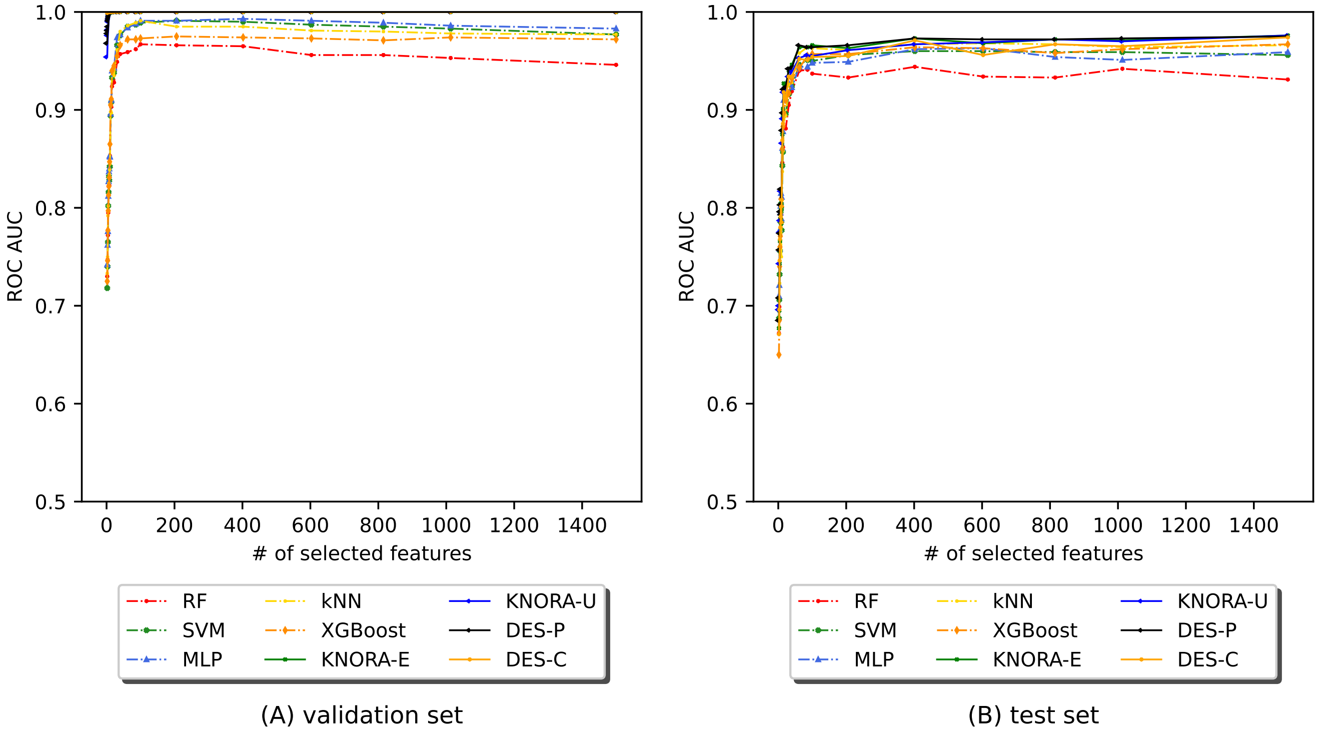

Figure S4. Evolution of the AUC as a function a larger number of selected features (NSF) for different models applied to the DA dataset. The models were analysed using (A) cross-validation and, (B) the test set. The following models and ensembles used were RF, SVM, MLP, kNN, XGBoost, KNORA-E, KNORA-U, DES-P and DES-C.

Table S1. Bioprojects used to compose the periodontitis and gingivitis dataset.

BioProject	Collection site	Diagnosis	# samples
PRJNA477241	Plaque	Periodontitis	18
PRJEB17957	Plaque	Periodontitis	12
PRJEB18651	Plaque	Periodontitis	89
PRJNA579148	Plaque	Periodontitis	30
PRJNA725103	Plaque	Periodontitis	42
PRJNA690677	Plaque	Periodontitis	39
PRJNA379905	Plaque	Periodontitis	94
PRJDB9395	Plaque	Periodontitis	23
PRJNA416501	Plaque	Periodontitis	66
PRJNA517671	Plaque	Periodontitis	9
PRJNA664107	Plaque	Periodontitis	30
PRJNA494044	Plaque	Periodontitis	13
PRJNA576446	Plaque	Periodontitis	210
PRJNA625671	Plaque	Periodontitis	79
PRJNA625189	Plaque	Periodontitis	28
PRJNA625189	Plaque	Periodontitis	29
PRJNA649803	Plaque	Periodontitis	10
PRJNA649803	Plaque	Periodontitis	12
PRJEB28345	Plaque	Periodontitis	85
PRJEB28345	Plaque	Periodontitis	169
PRJNA601054	Saliva	Periodontitis	97
PRJEB21767	Saliva	Periodontitis	46
PRJNA623352	Saliva	Periodontitis	27
PRJNA471556	Saliva	Periodontitis	70
PRJNA471556	Plaque	Periodontitis	35
PRJNA509532	Plaque	Periodontitis	97
PRJNA580506	Plaque	Periodontitis	7
PRJNA580506	Plaque	Periodontitis	7
PRJNA773202	Plaque	Periodontitis	65
PRJNA774299	Saliva	Periodontitis	36
PRJNA774981	Saliva	Periodontitis	38
PRJNA494044	Plaque	Gingivitis	12
PRJNA650272	Plaque	Gingivitis	48
PRJNA649803	Plaque	Gingivitis	8
PRJNA649803	Plaque	Gingivitis	12
PRJEB11529	Saliva	Gingivitis	18
PRJEB11529	Plaque	Gingivitis	19
PRJEB17957	Plaque	Health	8
PRJNA579148	Plaque	Health	28
PRJNA664107	Plaque	Health	16
PRJNA494044	Plaque	Health	11
DOI1252799	Plaque	Health	5
PRJNA650272	Plaque	Health	16
PRJNA576446	Plaque	Health	62
PRJNA625671	Plaque	Health	32
PRJNA649803	Plaque	Health	12
PRJNA649803	Plaque	Health	18
PRJNA601054	Saliva	Health	27
PRJNA503603	Saliva	Health	80
PRJEB21767	Saliva	Health	47
PRJNA534340	Saliva	Health	120
PRJNA586723	Saliva	Health	60
PRJNA623352	Saliva	Health	17
PRJNA471556	Saliva	Health	4
PRJNA471556	Plaque	Health	2
PRJEB11529	Saliva	Health	78
PRJEB11529	Plaque	Health	76
PRJNA509532	Plaque	Health	16
PRJNA580506	Plaque	Health	7
PRJNA580506	Plaque	Health	7
PRJNA773202	Plaque	Health	53
PRJNA774299	Saliva	Health	22
PRJNA774981	Saliva	Health	28

Table S2. Hyperparameter tuning step: parameters evaluated for each model.*

Model	Parameter	Evaluated values
RF	n_estimators	[50, 100, 300]
	max_depth	[3, 5, 10]
	max_features	["sqrt", "log2"]
	min_samples_split	[2, 3, 5, 10]
	min_samples_leaf	[2, 3]
	criterion	["gini", "entropy"]
	class_weight	["balanced", None]
SVM	C	[1, 10, 100]
	kernel	["linear", "rbf", "poly"]
	gamma	["scale", "auto", 0.1, 1, 10, 100]
	degree	[2, 3, 4]
MLP	hidden_layer_sizes	[(128,64,32), (64,32,16), (128,64), (64,32), (32, 16), (16, 8), (16), (8)]
	tol	[0.0001, 0.001, 0.01, 0.1, 1]
	alpha	[0.0001, 0.001, 0.01, 0.1, 1]
	n_iter_no_change	[10, 20, 30, 40, 50]
	max_iter	[500,1000]
	solver	["adam"]
	early_stopping	[True]
XGBoost	n_estimators	[60, 100, 140, 180, 220]
	max_depth	[2..10]
	learning_rate	[0.01, 0.05, 0.1, 0.15, 0.2]
	min_child_weight	[3, 5, 10]
kNN	n_neighbors	[1..31]

* Parameter descriptions and possible values can be consulted in the documentation of the Scikit-learn library, available at: `https://scikit-learn.org/stable/user_guide.html`

Table S3. Target number of features in comparison to the final selected features and the contribution of each method. NSF: number of selected features in common. PD_s: saliva samples with periodontal disease and healthy. PGD_s: saliva samples with periodontal disease, gingivitis, and healthy. PD_p: plaque samples with periodontal disease and healthy. PGD_p: plaque samples with periodontal disease, gingivitis, and healthy. RFE: Recursive Feature Elimination. RF: Random Forest. SVM: Support Vector Machine. LR: Logistic Regression.

	PD_s			PGD_s			PD_p			PGD_p		
NSF	RFE-RF	RFE-SVM	RFE-LR	RFE-RF	RFE-SVM	RFE-LR	RFE-RF	RFE-SVM	RFE-LR	RFE-RF	RFE-SVM	RFE-LR
15	32	64	69	-	-	-	-	-	-	-	-	-
14	31	60	65	29	59	58	31	56	53	21	45	48
13	30	58	63	27	55	57	29	54	52	20	43	46
12	29	55	60	26	52	54	25	51	48	20	42	44
11	26	49	54	24	47	50	23	48	44	17	37	37
10	23	37	42	23	40	45	21	48	43	17	36	35
9	23	37	41	22	39	44	19	41	37	17	36	34
8	22	36	40	22	35	41	17	37	32	16	36	34
7	21	35	39	-	-	-	19	43	39	13	29	27
6	18	34	38	20	32	36	14	32	30	12	27	25
5	16	28	33	17	28	30	13	31	29	11	27	23
4	12	21	27	13	25	26	12	28	28	10	27	23
3	8	9	12	8	14	17	9	24	22	7	14	12
2	6	7	9	5	9	10	7	16	15	6	11	11

Table S4. PD_s validation scores for each technique, using 10-fold stratified cross-validation. Only models with over 8 features are shown, which correspond to Figure 4.A of the paper (for synthesis purposes, we excluded data for features 2 through 7).

NSF	Score	kNN	RF	SVM	MLP	XGBoost	KNORA-E	KNORA-U	DES-P	DES-C
15	f1	0.890	0.871	0.888	0.855	0.868	0.955	0.944	0.944	0.957
	p	0.890	0.836	0.853	0.861	0.851	0.946	0.929	0.929	0.942
	r	0.890	0.909	0.927	0.849	0.886	0.963	0.959	0.959	0.973
	accuracy	0.914	0.894	0.908	0.887	0.894	0.964	0.955	0.955	0.966
	roc_auc	0.959	0.967	0.963	0.943	0.968	0.996	0.994	0.995	0.997
14	f1	0.888	0.867	0.893	0.876	0.884	0.966	0.952	0.952	0.966
	p	0.890	0.844	0.887	0.866	0.882	0.968	0.946	0.946	0.964
	r	0.886	0.890	0.900	0.886	0.886	0.963	0.959	0.959	0.968
	accuracy	0.912	0.892	0.916	0.901	0.908	0.973	0.962	0.962	0.973
	roc_auc	0.966	0.965	0.962	0.961	0.968	0.996	0.994	0.995	0.996
13	f1	0.890	0.840	0.882	0.849	0.877	0.970	0.950	0.947	0.972
	p	0.894	0.830	0.858	0.839	0.860	0.972	0.954	0.950	0.981
	r	0.886	0.849	0.909	0.858	0.895	0.968	0.945	0.945	0.963
	accuracy	0.914	0.873	0.905	0.880	0.901	0.977	0.961	0.959	0.978
	roc_auc	0.940	0.961	0.954	0.936	0.959	0.998	0.991	0.995	0.999
12	f1	0.885	0.867	0.886	0.817	0.876	0.972	0.949	0.952	0.991
	p	0.893	0.844	0.865	0.863	0.863	0.977	0.958	0.963	0.991
	r	0.877	0.890	0.909	0.776	0.890	0.968	0.941	0.941	0.991
	accuracy	0.910	0.892	0.908	0.864	0.901	0.978	0.961	0.962	0.993
	roc_auc	0.944	0.962	0.954	0.936	0.962	0.997	0.991	0.995	0.999
11	f1	0.886	0.858	0.888	0.869	0.866	0.925	0.914	0.919	0.938
	p	0.886	0.835	0.853	0.858	0.860	0.919	0.902	0.910	0.936
	r	0.886	0.881	0.927	0.881	0.872	0.932	0.927	0.927	0.941
	accuracy	0.910	0.885	0.908	0.896	0.894	0.941	0.932	0.935	0.952
	roc_auc	0.961	0.961	0.954	0.953	0.958	0.988	0.983	0.987	0.990
10	f1	0.870	0.852	0.881	0.846	0.852	0.959	0.943	0.941	0.964
	p	0.868	0.837	0.836	0.852	0.837	0.959	0.937	0.933	0.955
	r	0.872	0.868	0.932	0.840	0.868	0.959	0.950	0.950	0.973
	accuracy	0.898	0.882	0.901	0.880	0.882	0.968	0.955	0.953	0.971
	roc_auc	0.931	0.958	0.953	0.933	0.957	0.996	0.988	0.994	0.998
9	f1	0.887	0.856	0.877	0.841	0.858	0.950	0.921	0.928	0.941
	p	0.879	0.844	0.873	0.850	0.848	0.946	0.914	0.919	0.929
	r	0.895	0.868	0.881	0.831	0.868	0.954	0.927	0.936	0.954
	accuracy	0.910	0.885	0.903	0.876	0.887	0.961	0.937	0.943	0.953
	roc_auc	0.951	0.958	0.950	0.942	0.949	0.991	0.973	0.987	0.994
8	f1	0.882	0.855	0.878	0.869	0.864	0.952	0.943	0.938	0.986
	p	0.893	0.838	0.857	0.861	0.857	0.954	0.941	0.936	0.991
	r	0.872	0.872	0.900	0.877	0.872	0.950	0.945	0.941	0.982
	accuracy	0.908	0.883	0.901	0.896	0.892	0.962	0.955	0.952	0.989
	roc_auc	0.952	0.960	0.945	0.944	0.955	0.996	0.991	0.995	0.999

Table S5. PGD_s validation scores for each technique, using 10-fold stratified cross validation. Only models with over 8 features are shown, which correspond to Figure 4.B of the paper (for synthesis purposes, we excluded data for features 2 through 7).

NSF	Score	kNN	RF	SVM	MLP	XGBoost	KNORA-E	KNORA-U	DES-P	DES-C
15	f1	0.880	0.850	0.876	0.845	0.863	0.968	0.953	0.949	0.966
	p	0.865	0.828	0.874	0.838	0.864	0.966	0.961	0.953	0.962
	r	0.895	0.873	0.878	0.852	0.861	0.970	0.945	0.945	0.970
	accuracy	0.899	0.873	0.897	0.871	0.887	0.974	0.962	0.958	0.972
	roc_auc	0.940	0.953	0.959	0.936	0.950	0.998	0.995	0.996	0.997
14	f1	0.869	0.847	0.879	0.880	0.857	0.945	0.926	0.926	0.956
	p	0.857	0.821	0.885	0.892	0.857	0.953	0.928	0.924	0.954
	r	0.882	0.873	0.873	0.869	0.857	0.937	0.924	0.928	0.958
	accuracy	0.890	0.870	0.901	0.903	0.882	0.955	0.939	0.939	0.963
	roc_auc	0.949	0.950	0.945	0.943	0.951	0.990	0.983	0.986	0.992
13	f1	0.859	0.854	0.878	0.872	0.854	0.964	0.939	0.932	0.981
	p	0.857	0.844	0.888	0.880	0.846	0.966	0.937	0.932	0.979
	r	0.861	0.865	0.869	0.865	0.861	0.962	0.941	0.932	0.983
	accuracy	0.883	0.878	0.901	0.896	0.878	0.970	0.950	0.944	0.984
	roc_auc	0.942	0.955	0.946	0.948	0.949	0.995	0.990	0.992	0.997
12	f1	0.857	0.836	0.885	0.835	0.863	0.926	0.911	0.908	0.956
	p	0.857	0.813	0.879	0.858	0.849	0.932	0.911	0.904	0.943
	r	0.857	0.861	0.890	0.814	0.878	0.920	0.911	0.911	0.970
	accuracy	0.882	0.861	0.904	0.868	0.885	0.939	0.927	0.923	0.963
	roc_auc	0.943	0.950	0.943	0.925	0.947	0.986	0.977	0.983	0.990
11	f1	0.857	0.857	0.866	0.839	0.865	0.968	0.947	0.939	0.983
	p	0.854	0.833	0.872	0.803	0.852	0.966	0.945	0.937	0.979
	r	0.861	0.882	0.861	0.878	0.878	0.970	0.949	0.941	0.987
	accuracy	0.882	0.878	0.890	0.861	0.887	0.974	0.957	0.950	0.986
	roc_auc	0.933	0.954	0.943	0.920	0.947	0.997	0.988	0.993	0.999
10	f1	0.846	0.849	0.860	0.834	0.851	0.966	0.924	0.914	0.996
	p	0.847	0.831	0.867	0.851	0.846	0.974	0.928	0.908	0.992
	r	0.844	0.869	0.852	0.819	0.857	0.958	0.920	0.920	1.000
	accuracy	0.873	0.873	0.885	0.866	0.877	0.972	0.937	0.929	0.997
	roc_auc	0.937	0.949	0.936	0.915	0.947	0.996	0.983	0.990	1.000
9	f1	0.838	0.838	0.847	0.798	0.842	0.945	0.928	0.926	0.981
	p	0.848	0.824	0.864	0.812	0.831	0.953	0.932	0.928	0.971
	r	0.827	0.852	0.831	0.785	0.852	0.937	0.924	0.924	0.992
	accuracy	0.868	0.864	0.877	0.837	0.868	0.955	0.941	0.939	0.984
	roc_auc	0.927	0.938	0.928	0.901	0.945	0.992	0.977	0.988	0.997
8	f1	0.846	0.816	0.858	0.845	0.838	0.951	0.921	0.917	0.977
	p	0.857	0.793	0.887	0.850	0.836	0.961	0.931	0.927	0.967
	r	0.835	0.840	0.831	0.840	0.840	0.941	0.911	0.907	0.987
	accuracy	0.875	0.843	0.887	0.873	0.866	0.960	0.936	0.932	0.981
	roc_auc	0.925	0.937	0.927	0.931	0.939	0.995	0.975	0.986	0.998

Table S6. PD_p validation scores for each technique, using 10-fold stratified cross validation. Only models with over 8 features are shown, which correspond to Figure 4.C of the paper (for synthesis purposes, we excluded data for features 2 through 7).

NSF	Score	kNN	RF	SVM	MLP	XGBoost	KNORA-E	KNORA-U	DES-P	DES-C
15	f1	0.922	0.919	0.927	0.919	0.919	0.957	0.946	0.942	0.964
	p	0.899	0.891	0.915	0.905	0.893	0.954	0.944	0.940	0.955
	r	0.947	0.950	0.940	0.932	0.947	0.959	0.948	0.943	0.972
	accuracy	0.876	0.871	0.886	0.872	0.871	0.956	0.946	0.942	0.963
	roc_auc	0.908	0.911	0.900	0.907	0.918	0.991	0.988	0.988	0.993
14	f1	0.921	0.924	0.925	0.917	0.922	0.953	0.939	0.938	0.961
	p	0.892	0.897	0.909	0.904	0.885	0.949	0.934	0.936	0.950
	r	0.952	0.951	0.942	0.930	0.964	0.956	0.943	0.940	0.973
	accuracy	0.873	0.878	0.882	0.869	0.874	0.952	0.938	0.938	0.961
	roc_auc	0.899	0.909	0.894	0.906	0.907	0.988	0.983	0.986	0.992
13	f1	0.918	0.925	0.922	0.920	0.922	0.951	0.939	0.941	0.955
	p	0.898	0.899	0.903	0.908	0.902	0.945	0.939	0.942	0.946
	r	0.939	0.954	0.941	0.932	0.943	0.957	0.940	0.940	0.964
	accuracy	0.870	0.880	0.876	0.874	0.877	0.951	0.939	0.941	0.954
	roc_auc	0.890	0.906	0.901	0.909	0.915	0.988	0.982	0.986	0.991
12	f1	0.911	0.916	0.921	0.916	0.921	0.925	0.917	0.916	0.948
	p	0.886	0.883	0.901	0.890	0.903	0.919	0.913	0.909	0.933
	r	0.937	0.951	0.942	0.943	0.939	0.932	0.922	0.923	0.963
	accuracy	0.858	0.865	0.875	0.866	0.874	0.925	0.917	0.915	0.947
	roc_auc	0.876	0.893	0.898	0.883	0.909	0.982	0.973	0.977	0.988
11	f1	0.910	0.920	0.920	0.913	0.918	0.930	0.924	0.920	0.952
	p	0.891	0.887	0.895	0.897	0.896	0.915	0.903	0.901	0.938
	r	0.930	0.956	0.947	0.930	0.941	0.946	0.946	0.940	0.966
	accuracy	0.858	0.872	0.873	0.863	0.870	0.929	0.922	0.919	0.951
	roc_auc	0.861	0.894	0.884	0.876	0.901	0.983	0.977	0.979	0.988
10	f1	0.902	0.913	0.911	0.909	0.916	0.914	0.919	0.916	0.938
	p	0.885	0.884	0.880	0.898	0.898	0.905	0.911	0.908	0.924
	r	0.921	0.945	0.945	0.921	0.934	0.923	0.926	0.924	0.951
	accuracy	0.845	0.861	0.857	0.858	0.866	0.913	0.918	0.915	0.937
	roc_auc	0.849	0.886	0.873	0.876	0.886	0.978	0.977	0.977	0.985
9	f1	0.902	0.913	0.913	0.911	0.914	0.906	0.905	0.905	0.931
	p	0.881	0.884	0.880	0.893	0.894	0.892	0.898	0.897	0.917
	r	0.923	0.943	0.948	0.929	0.934	0.921	0.912	0.913	0.946
	accuracy	0.844	0.860	0.859	0.859	0.863	0.905	0.904	0.904	0.930
	roc_auc	0.846	0.878	0.868	0.866	0.881	0.973	0.969	0.970	0.979
8	f1	0.904	0.907	0.913	0.908	0.912	0.859	0.864	0.860	0.878
	p	0.889	0.866	0.882	0.895	0.882	0.840	0.845	0.840	0.850
	r	0.920	0.952	0.946	0.922	0.943	0.879	0.884	0.880	0.907
	accuracy	0.849	0.849	0.859	0.855	0.859	0.856	0.861	0.856	0.874
	roc_auc	0.854	0.858	0.863	0.870	0.873	0.941	0.937	0.939	0.947

Table S7. PGD_p validation scores for each technique, using 10-fold stratified cross validation. Only models with over 8 features are shown, which correspond to Figure 4.D of the paper (for synthesis purposes, we excluded data for features 2 through 7).

NSF	Score	kNN	RF	SVM	MLP	XGBoost	KNORA-E	KNORA-U	DES-P	DES-C
15	f1	0.921	0.919	0.929	0.922	0.921	0.942	0.937	0.937	0.961
	p	0.899	0.916	0.908	0.911	0.915	0.939	0.938	0.940	0.954
	r	0.944	0.923	0.952	0.933	0.928	0.945	0.937	0.934	0.970
	accuracy	0.872	0.872	0.886	0.875	0.875	0.942	0.937	0.937	0.961
	roc_auc	0.885	0.915	0.883	0.903	0.913	0.989	0.986	0.987	0.993
14	f1	0.919	0.918	0.927	0.921	0.923	0.923	0.905	0.907	0.932
	p	0.894	0.889	0.907	0.914	0.904	0.924	0.908	0.909	0.924
	r	0.945	0.950	0.949	0.928	0.944	0.922	0.901	0.904	0.941
	accuracy	0.868	0.867	0.882	0.874	0.877	0.923	0.905	0.907	0.931
	roc_auc	0.887	0.908	0.893	0.902	0.906	0.977	0.967	0.973	0.982
13	f1	0.914	0.920	0.923	0.915	0.911	0.962	0.933	0.930	0.981
	p	0.895	0.892	0.897	0.906	0.901	0.962	0.934	0.931	0.974
	r	0.934	0.950	0.951	0.924	0.922	0.962	0.932	0.929	0.988
	accuracy	0.862	0.869	0.875	0.865	0.858	0.962	0.933	0.929	0.981
	roc_auc	0.887	0.901	0.885	0.900	0.895	0.995	0.981	0.987	0.998
12	f1	0.918	0.914	0.923	0.914	0.917	0.925	0.908	0.909	0.936
	p	0.890	0.886	0.892	0.904	0.899	0.922	0.913	0.918	0.927
	r	0.946	0.943	0.956	0.924	0.935	0.928	0.903	0.900	0.945
	accuracy	0.866	0.859	0.874	0.863	0.866	0.924	0.908	0.909	0.935
	roc_auc	0.881	0.895	0.878	0.895	0.900	0.978	0.966	0.973	0.984
11	f1	0.911	0.914	0.918	0.908	0.916	0.926	0.915	0.912	0.932
	p	0.887	0.890	0.898	0.881	0.887	0.922	0.916	0.915	0.929
	r	0.937	0.939	0.939	0.937	0.947	0.930	0.914	0.909	0.935
	accuracy	0.856	0.860	0.867	0.851	0.863	0.925	0.915	0.912	0.931
	roc_auc	0.883	0.886	0.860	0.849	0.891	0.979	0.971	0.974	0.981
10	f1	0.910	0.911	0.907	0.904	0.905	0.892	0.889	0.891	0.877
	p	0.889	0.859	0.883	0.892	0.873	0.887	0.894	0.886	0.867
	r	0.932	0.968	0.932	0.916	0.939	0.898	0.884	0.896	0.888
	accuracy	0.854	0.850	0.849	0.846	0.844	0.891	0.889	0.890	0.875
	roc_auc	0.857	0.875	0.864	0.877	0.876	0.960	0.957	0.959	0.959
9	f1	0.910	0.908	0.907	0.901	0.908	0.910	0.895	0.897	0.933
	p	0.895	0.857	0.884	0.895	0.849	0.907	0.909	0.905	0.942
	r	0.924	0.964	0.931	0.908	0.975	0.913	0.881	0.889	0.924
	accuracy	0.855	0.845	0.849	0.843	0.843	0.909	0.896	0.897	0.934
	roc_auc	0.867	0.871	0.859	0.864	0.859	0.975	0.965	0.970	0.981
8	f1	0.907	0.907	0.909	0.903	0.912	0.869	0.867	0.865	0.881
	p	0.894	0.858	0.882	0.885	0.871	0.866	0.878	0.870	0.885
	r	0.920	0.963	0.938	0.921	0.958	0.873	0.856	0.861	0.876
	accuracy	0.851	0.845	0.853	0.843	0.855	0.868	0.868	0.866	0.881
	roc_auc	0.855	0.866	0.856	0.862	0.866	0.948	0.945	0.949	0.955

Table S8. F1 score (f1), precision (p), recall (r), accuracy (acc), and area under the curve (roc_auc) for each algorithm evaluated for the periodontitis subsets. Average of the validation scores over the different number of features (from 2 to 15).

Subset	Algorithms	f1	p	r	acc	roc_auc
PD_s	RF	0.888	0.856	0.924	0.909	0.963
	SVM	0.876	0.848	0.907	0.899	0.954
	kNN	0.875	0.880	0.870	0.902	0.956
	MLP	0.868	0.857	0.885	0.895	0.952
	XGBoost	0.874	0.865	0.883	0.900	0.963
	DES-C ensemble	0.885	0.869	0.902	0.908	0.964
	DES-P ensemble	0.891	0.874	0.908	0.913	0.966
	KNORA-E ensemble	0.878	0.871	0.885	0.903	0.964
	KNORA-U ensemble	0.890	0.875	0.907	0.912	0.963
PGD_s	RF	0.857	0.794	0.931	0.878	0.954
	SVM	0.848	0.825	0.873	0.877	0.933
	kNN	0.835	0.813	0.859	0.867	0.932
	MLP	0.848	0.822	0.880	0.877	0.934
	XGBoost	0.857	0.821	0.896	0.882	0.948
	DES-C ensemble	0.850	0.810	0.895	0.876	0.949
	DES-P ensemble	0.858	0.826	0.893	0.884	0.952
	KNORA-E ensemble	0.852	0.817	0.890	0.879	0.948
	KNORA-U ensemble	0.863	0.831	0.898	0.888	0.952
PD_p	RF	0.880	0.873	0.887	0.813	0.828
	SVM	0.851	0.893	0.822	0.783	0.820
	kNN	0.864	0.875	0.855	0.792	0.811
	MLP	0.856	0.896	0.826	0.789	0.826
	XGBoost	0.871	0.881	0.866	0.804	0.832
	DES-C ensemble	0.876	0.876	0.876	0.808	0.834
	DES-P ensemble	0.877	0.885	0.869	0.810	0.838
	KNORA-E ensemble	0.878	0.884	0.873	0.813	0.837
	KNORA-U ensemble	0.875	0.887	0.865	0.810	0.839
PGD_p	RF	0.881	0.887	0.876	0.814	0.814
	SVM	0.861	0.908	0.822	0.792	0.814
	kNN	0.864	0.901	0.832	0.794	0.809
	MLP	0.855	0.906	0.811	0.784	0.813
	XGBoost	0.877	0.891	0.864	0.810	0.816
	DES-C ensemble	0.876	0.892	0.862	0.809	0.823
	DES-P ensemble	0.875	0.903	0.849	0.809	0.824
	KNORA-E ensemble	0.877	0.895	0.861	0.810	0.819
	KNORA-U ensemble	0.874	0.905	0.847	0.808	0.825

Table S9. Results of the Venkatraman test comparing ensembles vs. individual models for the PD dataset, grouped by feature size.

Dataset	Subset	Evaluation[1]	Feature Group[2]	Total Tests[3]	Significant[4]	% Significant[5]
Periodontitis	PD_s	CV	Large	140	140	100.00
		CV	Small	280	280	100.00
		Test	Large	140	31	22.14
		Test	Small	280	36	12.86
Periodontitis	PGD_s	CV	Large	140	140	100.00
		CV	Small	260	260	100.00
		Test	Large	140	40	28.57
		Test	Small	260	64	24.62
Periodontitis	PD_p	CV	Large	140	140	100.00
		CV	Small	280	279	99.64
		Test	Large	140	72	51.43
		Test	Small	280	75	26.79
Periodontitis	PGD_p	CV	Large	140	140	100.00
		CV	Small	280	276	98.57
		Test	Large	140	68	48.57
		Test	Small	280	35	12.50

[1]Evaluation type: CV = cross-validation; Test = independent test set.
[2]Groups based on number of selected features."Small": < 100 features; 'Large": ≥ 100 features.
[3]Total number of Venkatraman tests performed in each setting.
[4]Number of tests where ensemble models significantly outperformed individual models ($p < 0.05$).
[5]Percentage of significant results = (Significant / Total Tests) $\times$ 100.

Table S10. PD_s test scores for the base models and the selected ensemble, DES-P. Only models with over 8 features are shown, which correspond to Figure 5.A of the paper (for synthesis purposes, we excluded data for features 2 through 7).

NSF	Score	kNN	RF	SVM	MLP	XGBoost	DES-P
15	f1	0.879	0.919	0.887	0.879	0.883	0.898
	p	0.909	0.875	0.860	0.837	0.883	0.903
	r	0.851	0.968	0.915	0.926	0.883	0.894
	accuracy	0.908	0.933	0.908	0.900	0.908	0.921
	roc_auc	0.971	0.970	0.971	0.953	0.974	0.974
14	f1	0.880	0.897	0.892	0.888	0.874	0.903
	p	0.900	0.870	0.902	0.892	0.865	0.913
	r	0.862	0.926	0.883	0.883	0.883	0.894
	accuracy	0.908	0.916	0.916	0.912	0.900	0.925
	roc_auc	0.970	0.971	0.971	0.970	0.972	0.978
13	f1	0.892	0.913	0.863	0.883	0.868	0.913
	p	0.902	0.881	0.825	0.845	0.863	0.881
	r	0.883	0.947	0.904	0.926	0.872	0.947
	accuracy	0.916	0.929	0.887	0.904	0.895	0.929
	roc_auc	0.954	0.969	0.963	0.953	0.966	0.973
12	f1	0.883	0.882	0.869	0.890	0.868	0.885
	p	0.883	0.851	0.827	0.876	0.863	0.867
	r	0.883	0.915	0.915	0.904	0.872	0.904
	accuracy	0.908	0.904	0.891	0.912	0.895	0.908
	roc_auc	0.954	0.969	0.962	0.958	0.968	0.972
11	f1	0.878	0.888	0.876	0.875	0.878	0.902
	p	0.874	0.853	0.822	0.857	0.874	0.879
	r	0.883	0.926	0.936	0.894	0.883	0.926
	accuracy	0.904	0.908	0.895	0.900	0.904	0.921
	roc_auc	0.962	0.972	0.968	0.956	0.972	0.973
10	f1	0.888	0.891	0.881	0.848	0.877	0.912
	p	0.892	0.869	0.824	0.867	0.882	0.889
	r	0.883	0.915	0.947	0.830	0.872	0.936
	accuracy	0.912	0.912	0.900	0.883	0.904	0.929
	roc_auc	0.947	0.964	0.964	0.948	0.965	0.967
9	f1	0.889	0.918	0.911	0.902	0.899	0.921
	p	0.884	0.890	0.897	0.879	0.895	0.907
	r	0.894	0.947	0.926	0.926	0.904	0.936
	accuracy	0.912	0.933	0.929	0.921	0.921	0.937
	roc_auc	0.954	0.966	0.962	0.965	0.965	0.969
8	f1	0.873	0.908	0.897	0.847	0.885	0.890
	p	0.908	0.873	0.870	0.842	0.867	0.876
	r	0.840	0.947	0.926	0.851	0.904	0.904
	accuracy	0.904	0.925	0.916	0.879	0.908	0.912
	roc_auc	0.953	0.962	0.971	0.937	0.963	0.969

Table S11. PGD_s test scores for the base models and the selected ensemble, DES-P. Only models with over 8 features are shown, which correspond to Figure 5.B of the paper (for synthesis purposes, we excluded data for features 2 through 7).

NSF	Score	kNN	RF	SVM	MLP	XGBoost	DES-P
15	f1	0.845	0.863	0.860	0.872	0.879	0.859
	p	0.820	0.800	0.838	0.842	0.837	0.817
	r	0.872	0.936	0.883	0.904	0.926	0.904
	accuracy	0.874	0.883	0.887	0.895	0.900	0.883
	roc_auc	0.917	0.956	0.946	0.945	0.958	0.953
14	f1	0.837	0.860	0.853	0.859	0.884	0.867
	p	0.804	0.788	0.816	0.817	0.838	0.833
	r	0.872	0.947	0.894	0.904	0.936	0.904
	accuracy	0.866	0.879	0.879	0.883	0.904	0.891
	roc_auc	0.936	0.956	0.942	0.939	0.955	0.957
13	f1	0.850	0.866	0.857	0.854	0.882	0.883
	p	0.802	0.813	0.824	0.810	0.851	0.845
	r	0.904	0.926	0.894	0.904	0.915	0.926
	accuracy	0.874	0.887	0.883	0.879	0.904	0.904
	roc_auc	0.930	0.958	0.939	0.938	0.961	0.957
12	f1	0.845	0.868	0.864	0.866	0.869	0.873
	p	0.820	0.802	0.819	0.813	0.827	0.835
	r	0.872	0.947	0.915	0.926	0.915	0.915
	accuracy	0.874	0.887	0.887	0.887	0.891	0.895
	roc_auc	0.936	0.959	0.948	0.948	0.960	0.959
11	f1	0.843	0.861	0.860	0.854	0.863	0.874
	p	0.806	0.806	0.811	0.810	0.825	0.829
	r	0.883	0.926	0.915	0.904	0.904	0.926
	accuracy	0.870	0.883	0.883	0.879	0.887	0.895
	roc_auc	0.930	0.960	0.944	0.943	0.955	0.957
10	f1	0.850	0.866	0.862	0.876	0.838	0.862
	p	0.828	0.813	0.832	0.822	0.798	0.832
	r	0.872	0.926	0.894	0.936	0.883	0.894
	accuracy	0.879	0.887	0.887	0.895	0.866	0.887
	roc_auc	0.948	0.958	0.941	0.941	0.938	0.957
9	f1	0.866	0.848	0.862	0.872	0.854	0.854
	p	0.840	0.808	0.832	0.842	0.810	0.837
	r	0.894	0.894	0.894	0.904	0.904	0.872
	accuracy	0.891	0.874	0.887	0.895	0.879	0.883
	roc_auc	0.948	0.953	0.941	0.940	0.942	0.958
8	f1	0.860	0.873	0.865	0.862	0.822	0.862
	p	0.838	0.809	0.847	0.832	0.786	0.832
	r	0.883	0.947	0.883	0.894	0.862	0.894
	accuracy	0.887	0.891	0.891	0.887	0.854	0.887
	roc_auc	0.938	0.962	0.938	0.940	0.933	0.952

Table S12. PD_p test scores for the base models and the selected ensemble, DES-P. Only models with over 8 features are shown, which correspond to Figure 5.C of the paper (for synthesis purposes, we excluded data for features 2 through 7).

NSF	Score	kNN	RF	SVM	MLP	XGBoost	KNORA-E
15	f1	0.866	0.907	0.886	0.888	0.906	0.904
	p	0.898	0.895	0.899	0.916	0.896	0.909
	r	0.836	0.921	0.873	0.862	0.915	0.899
	accuracy	0.799	0.855	0.826	0.832	0.852	0.852
	roc_auc	0.867	0.891	0.868	0.885	0.898	0.901
14	f1	0.866	0.922	0.892	0.894	0.898	0.913
	p	0.898	0.904	0.902	0.931	0.887	0.924
	r	0.836	0.942	0.881	0.860	0.910	0.902
	accuracy	0.799	0.877	0.834	0.842	0.840	0.867
	roc_auc	0.874	0.901	0.871	0.885	0.888	0.903
13	f1	0.871	0.906	0.879	0.888	0.901	0.903
	p	0.918	0.886	0.907	0.918	0.900	0.918
	r	0.828	0.926	0.852	0.860	0.902	0.889
	accuracy	0.809	0.850	0.818	0.832	0.846	0.852
	roc_auc	0.869	0.897	0.876	0.874	0.900	0.897
12	f1	0.881	0.901	0.887	0.885	0.907	0.892
	p	0.901	0.896	0.913	0.920	0.912	0.909
	r	0.862	0.907	0.862	0.852	0.902	0.876
	accuracy	0.820	0.846	0.830	0.828	0.857	0.836
	roc_auc	0.866	0.880	0.866	0.876	0.898	0.894
11	f1	0.893	0.906	0.887	0.902	0.897	0.903
	p	0.905	0.907	0.913	0.887	0.897	0.898
	r	0.881	0.905	0.862	0.918	0.897	0.907
	accuracy	0.836	0.855	0.830	0.846	0.840	0.848
	roc_auc	0.861	0.884	0.862	0.865	0.883	0.885
10	f1	0.880	0.896	0.873	0.877	0.893	0.890
	p	0.887	0.901	0.894	0.916	0.894	0.904
	r	0.873	0.892	0.852	0.841	0.892	0.876
	accuracy	0.816	0.840	0.807	0.818	0.834	0.832
	roc_auc	0.828	0.869	0.851	0.860	0.874	0.872
9	f1	0.886	0.895	0.868	0.874	0.889	0.892
	p	0.897	0.901	0.896	0.909	0.898	0.902
	r	0.876	0.889	0.841	0.841	0.881	0.881
	accuracy	0.826	0.838	0.801	0.811	0.830	0.834
	roc_auc	0.825	0.858	0.842	0.849	0.862	0.859
8	f1	0.868	0.882	0.861	0.854	0.882	0.875
	p	0.887	0.867	0.888	0.900	0.894	0.890
	r	0.849	0.897	0.836	0.812	0.870	0.860
	accuracy	0.799	0.814	0.791	0.785	0.820	0.809
	roc_auc	0.823	0.816	0.835	0.831	0.838	0.843

Table S13. PGD_p test scores for the base models and the selected ensemble, DES-P. Only models with over 8 features are shown, which correspond to Figure 5.D of the paper (for synthesis purposes, we excluded data for features 2 through 7).

NSF	Score	kNN	RF	SVM	MLP	XGBoost	DES-P
15	f1	0.894	0.919	0.885	0.897	0.916	0.913
	p	0.920	0.917	0.935	0.920	0.919	0.929
	r	0.870	0.922	0.841	0.875	0.914	0.897
	accuracy	0.838	0.873	0.828	0.842	0.869	0.865
	roc_auc	0.871	0.897	0.883	0.875	0.894	0.897
14	f1	0.889	0.912	0.888	0.891	0.917	0.907
	p	0.928	0.914	0.937	0.933	0.923	0.933
	r	0.853	0.909	0.843	0.853	0.912	0.882
	accuracy	0.832	0.861	0.832	0.836	0.871	0.857
	roc_auc	0.878	0.891	0.882	0.885	0.889	0.895
13	f1	0.885	0.901	0.867	0.870	0.900	0.899
	p	0.925	0.908	0.940	0.938	0.916	0.927
	r	0.848	0.895	0.804	0.811	0.885	0.873
	accuracy	0.826	0.846	0.805	0.809	0.846	0.846
	roc_auc	0.881	0.889	0.883	0.877	0.895	0.895
12	f1	0.871	0.913	0.864	0.831	0.915	0.908
	p	0.918	0.914	0.934	0.938	0.919	0.933
	r	0.828	0.912	0.804	0.745	0.912	0.885
	accuracy	0.807	0.863	0.801	0.761	0.867	0.859
	roc_auc	0.861	0.888	0.872	0.854	0.886	0.885
11	f1	0.877	0.898	0.867	0.868	0.896	0.898
	p	0.929	0.897	0.916	0.930	0.905	0.922
	r	0.831	0.900	0.824	0.814	0.887	0.875
	accuracy	0.817	0.840	0.801	0.805	0.838	0.844
	roc_auc	0.862	0.866	0.847	0.857	0.874	0.877
10	f1	0.866	0.883	0.853	0.864	0.874	0.875
	p	0.911	0.894	0.930	0.927	0.891	0.926
	r	0.826	0.873	0.787	0.809	0.858	0.828
	accuracy	0.799	0.819	0.786	0.799	0.805	0.813
	roc_auc	0.828	0.840	0.840	0.859	0.850	0.853
9	f1	0.847	0.880	0.850	0.846	0.853	0.862
	p	0.927	0.886	0.928	0.896	0.899	0.912
	r	0.779	0.875	0.784	0.801	0.811	0.816
	accuracy	0.778	0.813	0.782	0.770	0.780	0.793
	roc_auc	0.836	0.844	0.836	0.803	0.836	0.845
8	f1	0.846	0.879	0.841	0.803	0.868	0.849
	p	0.898	0.886	0.926	0.924	0.891	0.903
	r	0.799	0.873	0.770	0.711	0.846	0.801
	accuracy	0.770	0.811	0.770	0.726	0.797	0.776
	roc_auc	0.800	0.835	0.822	0.817	0.832	0.829

Table S14. Provided as a CSV file in the supplementary material.

Table S15. Provided as a CSV file in the supplementary material.

Table S16. Provided as a CSV file in the supplementary material.

Table S17. Target number of features to achieve the desired final number of features (NSF) for the IBD dataset.

Dataset	NSF	Target number of features
IBD	6	73
	10	90
	14	110
	18	137
	22	150
	26	162
	30	184
	34	188
	38	200

Table S18. IBD validation scores for each technique, using 10-fold stratified cross-validation. Only models with over 10 features are shown, which correspond to Figure 6.A of the paper (for synthesis purposes, we excluded data for features 6 and 10).

NSF	Score	kNN	RF	SVM	MLP	XGBoost	KNORA-E	KNORA-U	DES-P	DES-C
38	f1	0.793	0.870	0.833	0.809	0.885	0.977	0.964	0.956	0.994
	p	0.804	0.842	0.857	0.828	0.883	0.975	0.969	0.963	0.989
	r	0.784	0.899	0.810	0.792	0.887	0.979	0.959	0.950	1.000
	accuracy	0.796	0.865	0.837	0.813	0.885	0.977	0.964	0.957	0.994
	roc_auc	0.858	0.936	0.900	0.887	0.947	0.998	0.991	0.994	0.999
34	f1	0.796	0.876	0.824	0.836	0.879	0.999	0.999	0.999	1.000
	p	0.794	0.862	0.848	0.838	0.880	0.999	0.997	0.997	1.000
	r	0.797	0.890	0.802	0.834	0.878	1.000	1.000	1.000	1.000
	accuracy	0.795	0.874	0.829	0.836	0.879	0.999	0.999	0.999	1.000
	roc_auc	0.862	0.937	0.893	0.906	0.946	1.000	1.000	1.000	1.000
30	f1	0.767	0.847	0.810	0.815	0.875	0.938	0.935	0.935	0.962
	p	0.787	0.823	0.840	0.819	0.863	0.941	0.948	0.947	0.947
	r	0.749	0.872	0.782	0.810	0.887	0.934	0.922	0.923	0.978
	accuracy	0.773	0.842	0.816	0.816	0.873	0.938	0.936	0.936	0.962
	roc_auc	0.840	0.925	0.879	0.884	0.939	0.982	0.974	0.981	0.989
26	f1	0.759	0.857	0.800	0.788	0.874	0.938	0.923	0.922	0.968
	p	0.784	0.825	0.827	0.802	0.869	0.938	0.935	0.930	0.950
	r	0.736	0.892	0.775	0.775	0.878	0.937	0.911	0.913	0.987
	accuracy	0.767	0.851	0.806	0.792	0.873	0.938	0.924	0.922	0.968
	roc_auc	0.834	0.932	0.876	0.865	0.942	0.982	0.972	0.978	0.990
22	f1	0.749	0.862	0.797	0.803	0.879	0.958	0.948	0.948	0.979
	p	0.765	0.833	0.826	0.801	0.870	0.952	0.951	0.950	0.965
	r	0.733	0.892	0.770	0.804	0.888	0.964	0.946	0.947	0.993
	accuracy	0.754	0.857	0.804	0.802	0.878	0.957	0.948	0.948	0.978
	roc_auc	0.825	0.934	0.870	0.871	0.941	0.989	0.984	0.986	0.993
18	f1	0.739	0.858	0.795	0.780	0.870	0.930	0.924	0.926	0.958
	p	0.732	0.831	0.818	0.791	0.852	0.918	0.924	0.926	0.938
	r	0.747	0.887	0.774	0.768	0.890	0.943	0.923	0.926	0.978
	accuracy	0.737	0.853	0.801	0.783	0.867	0.929	0.924	0.926	0.957
	roc_auc	0.819	0.925	0.874	0.857	0.932	0.981	0.979	0.980	0.987
14	f1	0.745	0.842	0.769	0.796	0.838	0.994	0.989	0.986	0.999
	p	0.734	0.831	0.772	0.790	0.833	0.992	0.985	0.981	0.999
	r	0.756	0.853	0.767	0.802	0.844	0.996	0.993	0.992	1.000
	accuracy	0.741	0.840	0.770	0.794	0.837	0.994	0.989	0.986	0.999
	roc_auc	0.800	0.915	0.830	0.863	0.915	1.000	0.998	0.999	1.000
10	f1	0.737	0.808	0.720	0.713	0.825	0.826	0.825	0.825	0.840
	p	0.698	0.741	0.717	0.724	0.778	0.817	0.821	0.815	0.793
	r	0.781	0.890	0.723	0.701	0.877	0.835	0.828	0.835	0.892
	accuracy	0.721	0.789	0.719	0.717	0.813	0.824	0.824	0.823	0.829
	roc_auc	0.792	0.879	0.782	0.771	0.889	0.902	0.900	0.900	0.913

Table S19. IBD test scores for the base models and the selected ensemble, DES-P. Only models with over 10 features are shown, which correspond to Figure 6.B of the paper (for synthesis purposes, we excluded data for features 6 and 10).

NSF	Score	kNN	RF	SVM	MLP	XGBoost	DES-P
38	f1	0.862	0.871	0.863	0.863	0.878	0.884
	p	0.919	0.831	0.913	0.922	0.855	0.899
	r	0.811	0.915	0.818	0.811	0.902	0.870
	accuracy	0.804	0.797	0.804	0.806	0.811	0.828
	roc_auc	0.857	0.816	0.866	0.857	0.836	0.871
34	f1	0.865	0.874	0.862	0.849	0.873	0.885
	p	0.887	0.845	0.916	0.884	0.854	0.883
	r	0.844	0.906	0.814	0.818	0.893	0.886
	accuracy	0.801	0.804	0.804	0.782	0.804	0.826
	roc_auc	0.820	0.814	0.860	0.827	0.823	0.863
30	f1	0.860	0.872	0.859	0.814	0.865	0.866
	p	0.932	0.836	0.925	0.891	0.845	0.893
	r	0.798	0.912	0.801	0.749	0.886	0.840
	accuracy	0.804	0.799	0.801	0.743	0.792	0.804
	roc_auc	0.860	0.813	0.853	0.815	0.834	0.852
26	f1	0.855	0.863	0.853	0.832	0.861	0.866
	p	0.934	0.833	0.924	0.908	0.846	0.890
	r	0.788	0.896	0.792	0.769	0.876	0.844
	accuracy	0.799	0.787	0.794	0.767	0.787	0.804
	roc_auc	0.851	0.825	0.854	0.846	0.834	0.859
22	f1	0.847	0.866	0.848	0.837	0.858	0.857
	p	0.923	0.828	0.917	0.865	0.841	0.877
	r	0.782	0.909	0.788	0.811	0.876	0.837
	accuracy	0.787	0.789	0.787	0.762	0.782	0.789
	roc_auc	0.836	0.808	0.835	0.803	0.820	0.836
18	f1	0.818	0.864	0.833	0.839	0.859	0.850
	p	0.883	0.829	0.918	0.884	0.848	0.868
	r	0.762	0.902	0.762	0.798	0.870	0.834
	accuracy	0.745	0.787	0.770	0.770	0.784	0.779
	roc_auc	0.771	0.806	0.819	0.826	0.801	0.821
14	f1	0.801	0.853	0.839	0.827	0.845	0.845
	p	0.839	0.830	0.896	0.865	0.807	0.854
	r	0.765	0.876	0.788	0.792	0.886	0.837
	accuracy	0.713	0.772	0.772	0.750	0.755	0.770
	roc_auc	0.728	0.759	0.819	0.795	0.737	0.792
10	f1	0.805	0.847	0.804	0.796	0.840	0.837
	p	0.838	0.800	0.877	0.884	0.794	0.844
	r	0.775	0.899	0.743	0.723	0.893	0.831
	accuracy	0.718	0.755	0.728	0.721	0.745	0.757
	roc_auc	0.754	0.749	0.784	0.780	0.747	0.773

Table S20. Results of the Venkatraman test comparing ensembles vs. individual models for the IBD dataset, grouped by feature size.

Dataset	Evaluation[1]	Feature Group[2]	Total Tests[3]	Significant[4]	% Significant[5]
IBD	CV	Large	120	120	100.00
	CV	Small	180	180	100.00
	Test	Large	120	72	60.00
	Test	Small	180	40	22.22

[1]Evaluation type: CV = cross-validation; Test = independent test set.
[2]Groups based on number of selected features."Small": $<$ 100 features; 'Large": $\geq$ 100 features.
[3]Total number of Venkatraman tests performed in each setting.
[4]Number of tests where ensemble models significantly outperformed individual models ($p < 0.05$).
[5]Percentage of significant results = (Significant / Total Tests) $\times$ 100.

Table S21. F1 score (f1), precision (p), recall (r), accuracy (acc) and area under the curve (roc_auc) for each algorithm evaluated for the IBD dataset. Average of the test scores over the different number of features (from 6 to 38).

Subset	Algorithms	f1	p	r	acc	roc_auc
IBD	RF	0.854	0.824	0.887	0.773	0.774
	SVM	0.838	0.895	0.788	0.769	0.809
	kNN	0.833	0.882	0.791	0.761	0.785
	MLP	0.828	0.875	0.787	0.754	0.796
	XGBoost	0.854	0.831	0.878	0.773	0.780
	DES-C ensemble	0.857	0.835	0.880	0.778	0.792
	DES-P ensemble	0.854	0.865	0.844	0.783	0.809
	KNORA-E ensemble	0.855	0.850	0.860	0.780	0.797
	KNORA-U ensemble	0.859	0.869	0.850	0.790	0.802

Table S22. Provided as a CSV file in the supplementary material.

Table S23. Provided as a CSV file in the supplementary material.

Table S24. DA validation scores for each technique, using 10-fold stratified cross-validation. Only models with over 7 features are shown, which correspond to Figure 7.A of the paper.

NSF	Score	kNN	RF	SVM	MLP	XGBoost	KNORA-E	KNORA-U	DES-P	DES-C
40	f1	0.927	0.891	0.921	0.917	0.905	1.000	1.000	1.000	1.000
	p	0.947	0.927	0.955	0.937	0.931	1.000	1.000	1.000	1.000
	r	0.908	0.857	0.889	0.899	0.880	1.000	1.000	1.000	1.000
	accuracy	0.928	0.895	0.923	0.919	0.907	1.000	1.000	1.000	1.000
	roc_auc	0.980	0.957	0.976	0.977	0.966	1.000	1.000	1.000	1.000
22	f1	0.877	0.854	0.869	0.864	0.857	0.998	0.998	0.996	1.000
	p	0.893	0.878	0.881	0.865	0.872	1.000	1.000	0.998	1.000
	r	0.861	0.832	0.857	0.863	0.842	0.996	0.996	0.994	1.000
	accuracy	0.879	0.858	0.871	0.864	0.859	0.998	0.998	0.996	1.000
	roc_auc	0.935	0.928	0.938	0.943	0.945	1.000	1.000	1.000	1.000
16	f1	0.869	0.855	0.852	0.858	0.854	0.998	0.997	0.997	1.000
	p	0.895	0.870	0.881	0.860	0.860	1.000	1.000	1.000	1.000
	r	0.845	0.840	0.826	0.855	0.849	0.996	0.994	0.994	1.000
	accuracy	0.873	0.857	0.857	0.858	0.855	0.998	0.997	0.997	1.000
	roc_auc	0.939	0.924	0.933	0.940	0.940	1.000	1.000	1.000	1.000
14	f1	0.830	0.821	0.841	0.832	0.823	0.993	0.993	0.992	1.000
	p	0.874	0.829	0.888	0.847	0.832	1.000	1.000	1.000	1.000
	r	0.790	0.813	0.798	0.817	0.813	0.985	0.985	0.983	1.000
	accuracy	0.838	0.822	0.848	0.835	0.824	0.993	0.993	0.992	1.000
	roc_auc	0.897	0.903	0.908	0.910	0.911	1.000	1.000	1.000	1.000
12	f1	0.822	0.812	0.817	0.806	0.813	0.994	0.987	0.988	1.000
	p	0.841	0.828	0.873	0.826	0.819	0.998	0.994	0.996	1.000
	r	0.803	0.796	0.767	0.788	0.807	0.989	0.981	0.981	1.000
	accuracy	0.825	0.815	0.827	0.811	0.814	0.994	0.987	0.988	1.000
	roc_auc	0.898	0.895	0.894	0.896	0.905	1.000	1.000	1.000	1.000
10	f1	0.751	0.788	0.736	0.773	0.781	0.983	0.978	0.969	1.000
	p	0.776	0.803	0.843	0.800	0.791	0.991	0.991	0.989	1.000
	r	0.727	0.773	0.653	0.748	0.771	0.975	0.964	0.950	1.000
	accuracy	0.758	0.792	0.765	0.780	0.783	0.983	0.978	0.969	1.000
	roc_auc	0.845	0.853	0.842	0.852	0.865	0.999	0.998	0.998	1.000
9	f1	0.762	0.763	0.729	0.754	0.754	0.985	0.970	0.965	1.000
	p	0.770	0.773	0.816	0.798	0.767	0.996	0.989	0.985	1.000
	r	0.754	0.752	0.660	0.714	0.742	0.975	0.952	0.945	1.000
	accuracy	0.764	0.765	0.755	0.766	0.758	0.985	0.971	0.965	1.000
	roc_auc	0.839	0.839	0.842	0.853	0.847	0.999	0.996	0.997	1.000
8	f1	0.746	0.748	0.749	0.762	0.745	0.978	0.958	0.953	1.000
	p	0.771	0.751	0.746	0.802	0.748	0.985	0.976	0.972	1.000
	r	0.723	0.746	0.752	0.725	0.742	0.971	0.941	0.935	1.000
	accuracy	0.754	0.748	0.747	0.773	0.745	0.978	0.959	0.954	1.000
	roc_auc	0.834	0.823	0.828	0.840	0.831	0.999	0.994	0.995	1.000

Table S25. DA test scores for the base models and the selected ensemble, DES-P. Only models with over 7 features are shown, which correspond to Figure 7.B of the paper.

NSF	Score	kNN	RF	SVM	MLP	XGBoost	DES-P
40	f1	0.848	0.806	0.843	0.849	0.838	0.858
	p	0.862	0.826	0.861	0.833	0.841	0.858
	r	0.835	0.787	0.827	0.866	0.835	0.858
	accuracy	0.885	0.855	0.882	0.882	0.876	0.891
	roc_auc	0.928	0.919	0.924	0.923	0.932	0.943
22	f1	0.806	0.748	0.789	0.774	0.777	0.814
	p	0.810	0.726	0.783	0.754	0.759	0.817
	r	0.803	0.772	0.795	0.795	0.795	0.811
	accuracy	0.852	0.801	0.837	0.822	0.825	0.858
	roc_auc	0.894	0.881	0.896	0.926	0.910	0.923
16	f1	0.772	0.755	0.733	0.785	0.773	0.780
	p	0.798	0.746	0.779	0.767	0.767	0.765
	r	0.748	0.764	0.693	0.803	0.780	0.795
	accuracy	0.831	0.810	0.807	0.831	0.825	0.828
	roc_auc	0.890	0.882	0.894	0.910	0.897	0.921
14	f1	0.724	0.707	0.719	0.739	0.727	0.748
	p	0.759	0.731	0.798	0.781	0.730	0.802
	r	0.693	0.685	0.654	0.701	0.724	0.701
	accuracy	0.798	0.782	0.804	0.810	0.792	0.819
	roc_auc	0.858	0.862	0.857	0.878	0.883	0.897
12	f1	0.718	0.742	0.701	0.704	0.739	0.757
	p	0.746	0.760	0.779	0.725	0.754	0.793
	r	0.693	0.724	0.638	0.685	0.724	0.724
	accuracy	0.792	0.807	0.792	0.779	0.804	0.822
	roc_auc	0.837	0.846	0.843	0.861	0.860	0.879
10	f1	0.677	0.658	0.645	0.655	0.648	0.625
	p	0.677	0.709	0.778	0.724	0.667	0.664
	r	0.677	0.614	0.551	0.598	0.630	0.591
	accuracy	0.752	0.755	0.767	0.758	0.737	0.728
	roc_auc	0.807	0.810	0.777	0.811	0.801	0.819
9	f1	0.617	0.651	0.631	0.646	0.628	0.617
	p	0.619	0.664	0.737	0.725	0.661	0.647
	r	0.614	0.638	0.551	0.583	0.598	0.591
	accuracy	0.707	0.737	0.752	0.755	0.728	0.719
	roc_auc	0.750	0.793	0.786	0.789	0.785	0.793
8	f1	0.661	0.648	0.654	0.684	0.656	0.667
	p	0.654	0.643	0.654	0.748	0.651	0.656
	r	0.669	0.654	0.654	0.630	0.661	0.677
	accuracy	0.737	0.728	0.734	0.776	0.734	0.740
	roc_auc	0.780	0.803	0.804	0.815	0.809	0.819

Table S26. F1 score (f1), precision (p), recall (r), accuracy (acc) and area under the curve (roc_auc) for each algorithm evaluated for the DA dataset. Average of the test scores over the different number of features (from 2 to 40).

Subset	Algorithms	f1	p	r	acc	roc_auc
DA	RF	0.664	0.667	0.662	0.742	0.808
	SVM	0.661	0.704	0.626	0.755	0.811
	kNN	0.680	0.691	0.670	0.758	0.800
	MLP	0.675	0.718	0.641	0.768	0.827
	XGBoost	0.673	0.669	0.678	0.746	0.813
	DES-C ensemble	0.686	0.684	0.690	0.756	0.820
	DES-P ensemble	0.683	0.694	0.673	0.760	0.831
	KNORA-E ensemble	0.687	0.684	0.692	0.757	0.823
	KNORA-U ensemble	0.694	0.699	0.689	0.766	0.826
